# Expression at the edge: Free speech boundaries amidst the Gaza crisis

**DOI:** 10.1126/sciadv.aea5427

**Published:** 2026-04-15

**Authors:** Ran Abramitzky, Guy Grossman, Yphtach Lelkes, Hani Mansour, Tamar Mitts

**Affiliations:** ^1^ Department of Economics, Stanford University, and NBER, Stanford, CA, USA.; ^2^ Department of Political Science, University of Pennsylvania, and PDRI-DevLab, Philadelphia, PA, USA.; ^3^ Annenberg School for Communication, University of Pennsylvania, Philadelphia, PA, USA.; ^4^ Department of Economics, University of Colorado Denver, Denver, CO, USA.; ^5^ School of International and Public Affairs, Columbia University, New York, NY, USA.

## Abstract

Universities have become key arenas in national debates over the boundaries of free expression. Using preregistered online survey experiments with a nationally representative sample of 3065 US college students, this study examines how individuals navigate the tension between free speech and harm prevention, an issue sharpened by recent campus protests over Gaza. We test how variation in the severity of speech and the identity of its target (white, Black, Jewish, Muslim, or transgender individuals) shapes judgments about appropriate institutional responses. Our preregistered analyses show that students generally oppose punishing objectionable speech unless it is perceived as highly harmful and that identical statements directed at minority groups elicit stronger punitive responses than those targeting white individuals. Exploratory analyses reveal that these patterns reflect distinct normative principles: Most students adopt a particularist stance, favoring greater protection for marginalized groups, while a sizable minority adhere to a universalist view emphasizing equal treatment regardless of identity. These principles predict attitudes across contexts, but adherence weakens when individuals hold strong views on the issue at hand. Our findings show that campus conflicts over speech boundaries reflect not only disagreement about norms but also unequal application of these norms across groups and issues.

## Teaser

Students draw campus speech lines using shared principles, yet perceived harm and ideological alignment shape who gets protected.

## INTRODUCTION

Few issues have polarized US campuses as sharply as the war in Gaza that followed the 7 October 2023 Hamas attacks and Israel’s military response. Student protests, counterprotests, and administrative interventions turned universities into highly visible battlegrounds over the boundaries of acceptable political expression. Campus debates focused heavily on whether particular slogans, chants, and statements were legitimate exercises of free speech or crossed a line into hateful or threatening speech that warranted punishment. At the same time, many protests involved encampments, building occupations, and physical blockades, blurring the line between speech and disruptive conduct. Here, we focus specifically on attitudes toward free speech: When people believe that objectionable speech should be permitted or punished, holding constant whether or not it is accompanied by disruptive behavior. The Gaza protests were not an isolated episode, but rather the latest flashpoint in an ongoing debate about the boundaries of acceptable speech on college campuses. From controversies over invited speakers to disputes about hate speech codes (e.g., the invalidation of the University of Michigan’s 1988 code in Doe v. University of Michigan), universities have repeatedly found themselves at the center of broader societal struggles over free expression and harm prevention (*1*–*3*). These conflicts have drawn national media attention, shaped political discourse, and even prompted congressional hearings and calls to defund major universities (*4*–*6*). In a way, campuses today function as microcosms of democratic societies, where competing visions of pluralism, equality, and tolerance are publicly contested. Understanding how college students interpret and apply free speech norms is therefore central to understanding how these broader conflicts emerge and evolve.

Our focus on college students is intentional rather than incidental. Students are not only participants in campus speech controversies but also key actors in shaping institutional norms. Their collective attitudes may influence university policy decisions, public perceptions of higher education, and, ultimately, the cultural boundaries of free expression itself. The university environment also serves as a formative arena where young adults encounter ideological diversity, navigate moral disagreement, and develop durable political and normative commitments. As legal scholars and theorists emphasize, campus speech debates carry implications far beyond university walls (*7*–*9*). Because campus controversies over speech help shape broader social understandings of what counts as acceptable expression, students’ views are indispensable for understanding how the next generation conceives the boundaries of free expression and the meaning of inclusion.

At the core of these debates lie two competing normative principles for regulating speech: universalism and particularism. The US Supreme Court has consistently rejected hate speech laws and campus speech codes, reasoning that they constitute viewpoint or content discrimination (*10*). This position reflects a universalist principle, which holds that all individuals deserve equal protection under the law, regardless of identity or status. Yet numerous legal scholars [e.g., (*11*–*13*)], as well as several liberal democracies such as Germany, Sweden, and Spain, have adopted a more particularist stance, recognizing that historically marginalized groups may require additional protections. For instance, the prohibition of Nazi symbols in parts of Europe acknowledges the specific harm that such speech inflicts on vulnerable communities (*14*, *15*).

This normative divide is mirrored in American political life. Conservative critiques of higher education frequently target what they see as the dominance of a particularist approach, most notably when speakers deemed harmful to marginalized groups are disinvited (*16*). Public opinion research further shows that, over the past two decades, educated liberals have become more accepting of speech restrictions justified as protecting minorities (*17*), while conservatives, who in the 1960s and 1970s were more likely to favor restrictions on speech opposing the Vietnam War, have become stronger defenders of unrestricted expression (*18*), especially online (*19*, *20*). Yet the Gaza protests scrambled these alignments: Many on the right demanded constraints on protesters’ speech, while many on the left invoked the principle of expressive freedom (*21*, *22*). This reversal illustrates that public judgments about dangerous or impermissible speech are highly context dependent, reflecting not only ideology but also the political and moral meaning attributed to specific events.

Despite intense attention to these debates, we still lack a systematic understanding of how people, and especially college students, draw the boundaries of protected expression. Prior surveys provide useful descriptive snapshots of support for free speech [e.g., (*23*, *24*)], but they rarely probe the mechanisms underlying these attitudes. For example, typical survey questions ask students whether they support restricting speech they find “offensive or biased,” without clarifying how responses depend on the identity of the target or the severity of the speech. Existing work therefore tells us little about how students balance competing principles of fairness and harm prevention or how their judgments vary across contexts and identities.

Our study addresses these gaps by focusing on two dimensions central to both legal and public debates about free expression: the identity of the speech target and the severity of the speech act. Specifically, respondents evaluated hypothetical statements made by either professors (the “professor experiment”) or students (the “student experiment”) that varied in (i) the identity of the targeted group (white, Black, Jewish, Muslim, or transgender individuals) and (ii) the severity of the speech (ranging from objectionable to explicitly hateful). We prioritized these attributes while holding others constant because they capture the central tensions at the heart of contemporary controversies, namely, which groups are targeted and when expression is perceived to cross the line from objectionable to harmful, while also maintaining realism and statistical power. In the professor experiment, we also varied the level of publicness of the speech to examine how context shapes reactions. By applying these manipulations to a student population directly embedded in the campus speech environment, we can assess whether patterns observed in the general public (*12*, *25*–*27*) replicate among those most directly engaged in these debates.

Consequently, our study is guided by three empirical questions. First, do students consider the target’s identity when evaluating whether speech deserves protection? Prior work offers mixed evidence on this point. Some studies suggest that the extremity of the speech content matters more than the identity of the target in shaping attitudes toward restricting speech (*19*, *25*), while others find that support for protecting speech varies systematically with the identity of targeted groups (*17*, *20*). Second, do students consciously endorse either universalistic or particularistic principles, and do they apply these principles consistently across cases? Last, do students’ stated commitments to democratic values such as free speech hold up under ideological cross-pressures, or do they weaken when judgments implicate their political identities? Research on democratic backsliding suggests that ideological attachments often override abstract principles: For example, Graham and Svolik (*28*) show that voters frequently prioritize partisan loyalty over democratic norms, while Chong *et al.* (*17*) demonstrate that attitudes toward speech restrictions often reflect ideological alignment more than stable principles. We extend these insights to the university setting, asking whether students’ stated commitments to universalism or particularism are genuine or strategically invoked to rationalize prior ideological positions.

To answer these questions, we conducted a series of online survey experiments in July 2024 with a nationally representative sample of 3065 college students. The analyses of the experimental outcomes were preregistered; analyses examining adherence to underlying principles were exploratory. Participants judged both the harm caused by the statements and the appropriate university response, ranging from no action to public condemnation to disciplinary measures such as suspension, firing, or expulsion. A separate policy experiment further assessed whether support for campus rules restricting offensive speech depends on which group the rule protects.

In the context of the Gaza protests, where free speech was often coupled with physical action on campus, our experiments were designed to measure respondents’ support for free speech even when it entails objectionable speech, rather than their tolerance for disruptive behavior. By separating attitudes toward speech from attitudes toward protest tactics, we clarify how students and adults think about the boundaries of free speech in a polarized and unequal society.

The core elements of our research design are three related experiments. The first experiment (“policy experiment”) captured students’ general support for speech restrictions as a means to protect target groups. The second experiment (professor experiment) assessed how students evaluate controversial statements made by a faculty member, varying both the targeted group and the severity of the speech. The third experiment (student experiment) mirrors the second experiment for statements made by another student, varying speech severity while limiting the targeted group to either Jews or Muslims.

Both the professor and student vignettes describe speech acts that occur in nondisruptive contexts, enabling us to observe how individuals delineate the boundary between objectionable and impermissible expression. Our design does not assume a fixed legal or procedural boundary between free speech and disruption; rather, it captures how individuals view this boundary within a realistic campus context. While real-world disputes about campus protests often combine procedural and expressive concerns, our study focuses more narrowly on how people evaluate contested speech itself.

We emphasize four key findings. First, consistent with our preregistered hypotheses, attitudes toward speech restrictions depend strongly on perceived harm severity and the identity of the targeted group. Second, most students adopt a particularist worldview, favoring greater protection for historically marginalized groups. However, while 64% endorse particularism, a substantial minority identify as universalists. Third, these principles, representing different ways of balancing free expression with inclusion and harm prevention, predict responses across scenarios beyond ideology and demographics. Fourth, these principles likely weaken when individuals hold strong views on the issue at stake. This fragility highlights a broader asymmetry: Campus conflicts over speech boundaries are not merely disagreements about abstract norms but reflections of unequal applications of those norms across groups and contexts. In the next section, we describe our experimental design and preregistered expectations.

## RESULTS

To estimate the effect of the treatments on the outcomes, we used ordinary least squares (OLS) regression models (see Materials and Methods). We first report preregistered results for the entire sample, unconditional on respondents’ speech boundary principles. We then turn to the exploratory results that examine whether support for speech punishment is (also) a function of a principled approach to possible limitations on freedom of speech.

### Speech harmfulness and target identity drive support for punishment and perceived harm

**Fig. 1.**
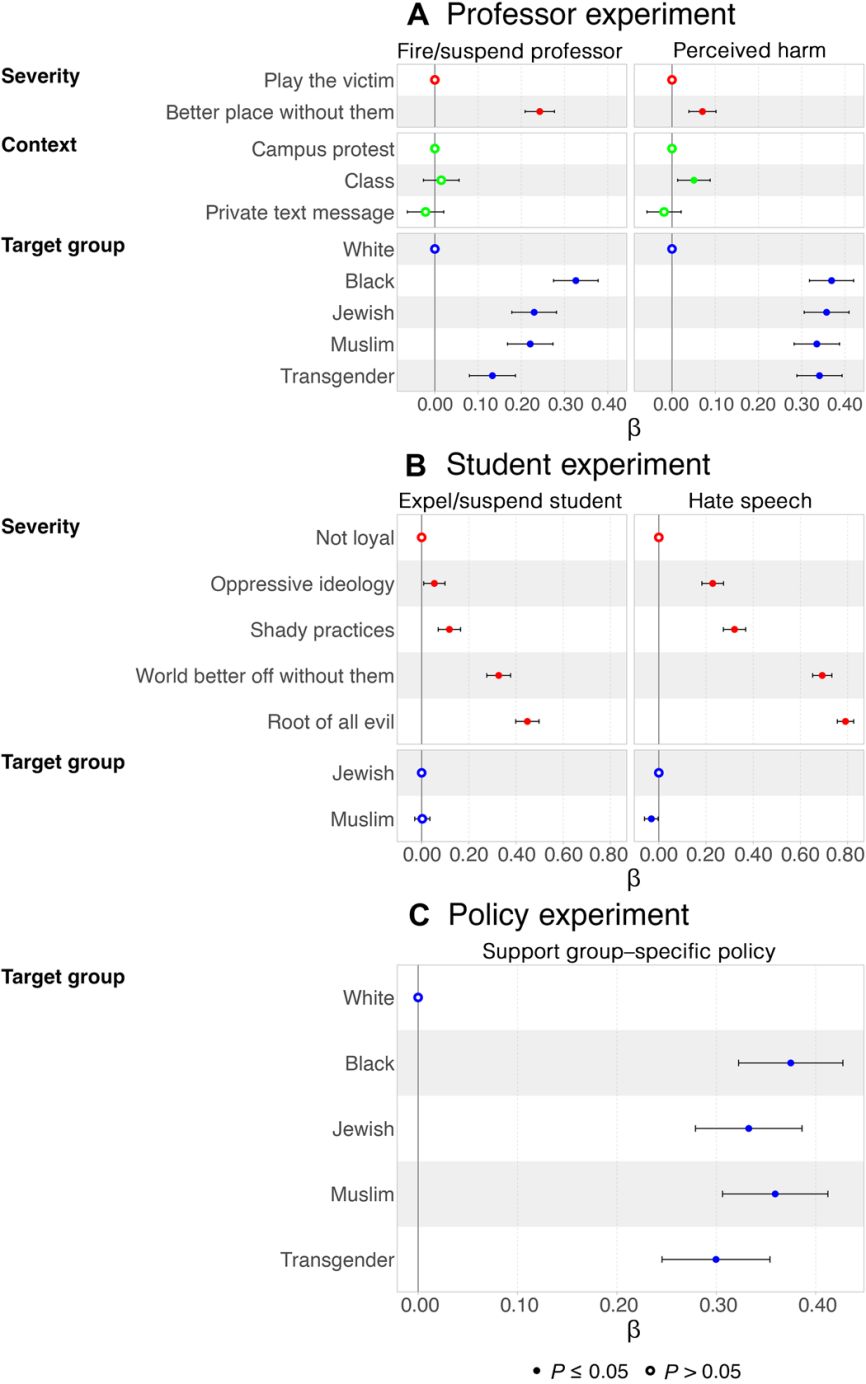
Support for speech restrictions varies by target identity and punishment severity. This figure presents results from three experiments assessing support for punishing speech on university campuses. The experiments involved randomized variations in the target group, speech severity, and the context of expression. Reference categories for each manipulation are marked by circles on the intercept line. (**A**) Professor experiment: left panel shows estimated effects on support for suspending or firing the professor; right panel shows estimated effects on the belief that the statement would cause physical or psychological harm. (**B**) Student experiment: left panel shows estimated effects on support for suspending or expelling the student; right panel shows estimated effects on whether the statement is classified as hate speech. (**C**) Policy experiment: estimated effects on support for endorsing a group-specific policy banning offensive public statements. Regression models are described in Materials and Methods. Full regression results in tabular form shown in tables S2 to S4.

Consistent with our preregistered hypotheses, we find that students are more likely to support suspending or firing the professor when the speech is perceived to be more harmful. Figure 1A presents coefficients from the professor experiment regression model outlined in Materials and Methods. The Supplementary Materials also reports the corresponding model-based predicted probabilities (rather than coefficients) for all figures (fig. S3 to S8). The outcome supporting suspending or firing the professor is displayed in the left panel, and the belief that the statement would cause physical or psychological harm is displayed in the right panel. Coefficients for severity appear in red. On average and pooled across all five possible target groups, 34% of respondents supported suspending or firing the professor when they asserted that the targeted group “play the victim to get special treatment” (the omitted category). Relative to this, saying the “US would be a better place without” the targeted group increases the probability of suspending or firing the professor by 24 percentage points (β = 0.24, *P* < 0.001; table S2). This increase is mirrored by perceptions that the “better place without them” statement causes more physical or psychological harm (β = 0.07, *P* < 0.001; table S2) compared to stating that the target group “play the victim.” While there is some evidence that the context of speech affected perceptions of harm, a statement that a professor made in class is perceived as more harmful than the same statement made in a private text message, we do not find that context affected support for punishment (coefficients related to speech context appear in green).

Similarly, in the student experiment, support for expelling or suspending the (fictitious) student depended on the severity of their statement. Figure 1B depicts the estimated coefficients based on the student experiment regression model outlined in Materials and Methods. Pooled across the two possible target groups (Jews and Muslims), about 17% of respondents support suspending or expelling students for describing the target group as “not loyal” (the omitted category). Relative to this reference statement, other statements elicited greater support for punishment, ranging from an increase of 5 percentage points (β = 0.05, *P* < 0.01; table S3) for attributing an “oppressive ideology” to the target group to an increase of 45 percentage points (β = 0.45, *P* < 0.001; table S3) for stating the target group is the “root of all evil.” Moreover, respondents were more likely to classify more severe statements as hate speech. About 11% of respondents considered not loyal to be hate speech, while 90% of respondents considered the root of all evil to be hate speech, representing an increase of 79 percentage points (β = 0.79, *P* < 0.001; table S3).

We also find that the identity of the target significantly shapes students’ responses (see blue dots in Fig. 1, A and B). Relative to harmful speech targeting white people, the probability of suspending or firing a professor making the same statement about Black people increases by 33 percentage points (β = 0.33, *P* < 0.001; table S2). Compared to white individuals, the probability of suspending or firing a professor for harmful speech targeted at Jewish or Muslim people increases by 23 (β = 0.23, *P* < 0.001; table S2) and 22 (β = 0.22, *P* < 0.001; table S2) percentage points, respectively. The effects for Jews and Muslim targets do not differ statistically. While the estimated coefficient for targeting transgender people is somewhat smaller (β = 0.13, *P* < 0.001), it is not statistically distinguishable from the estimated effects for Jewish or Muslim people. These results suggest that students do not treat harmful speech against Jewish and Muslim people differently in punishment judgements.

Although our results indicate that students are willing to provide stronger protections when the target group is Black people, they have similar assessments about whether statements would cause physical or psychological harm for all target groups relative to white people (Fig. 1A). This suggests that, while harm protection is the main channel tying group identity to speech restrictions, other considerations are likely present too. While support for punishing students in the student experiment did not vary when the target group was Jewish versus Muslim, we find that statements targeting Muslim individuals are slightly less likely to be perceived as hate speech compared to those targeting Jewish individuals, although the difference is relatively small (β = −0.03, *P* < 0.5; table S3). This is similar to findings from the professor experiment, in which students afforded Jewish and Muslim targets similar protection from harmful speech. This pattern should be interpreted as reflecting perceptions of marginalization rather than racial classification. In our framework, “group identity” captures multiple dimensions of vulnerability—including religion, ethnicity, and sexual orientation—rather than race alone. The timing of our study, amid heightened awareness of antisemitism and campus protests, may have further amplified recognition of Jewish students as a targeted group.

Last, we present results for our policy experiment in Fig. 1C (table S4). We find that, compared to white individuals (mean = 0.29), students were more likely to support a policy banning offensive public statements directed at Jewish (β = 0.33, *P* < 0.001) and Muslim (β = 0.36, *P* < 0.001) people. These effect sizes are not statistically different from those for Black (β = 0.38, *P* < 0.001) and transgender (β = 0.30, *P* < 0.001) individuals. These results suggest that the average US student sees both Jews and Muslims as minority groups, distinct from white Americans, and believes that they should receive similar levels of protection from harmful speech. Neither group is viewed as more deserving of protection than the other, while white Americans are afforded less concern in this regard. Notably, about 43% of respondents did not support a policy banning offensive public statements when the policy singles out a specific group for special treatment. We expand on the significance of this finding in the next section.

In exploratory analyses (reported in the Supplementary Materials), we assess heterogeneity by campus context. We compare respondents at institutions in predominantly Democratic (“blue”) versus Republican (“red”) states. The main treatment patterns are consistent across these subgroups, with only modest variation in magnitude. While the study was not powered for fine-grained geographic comparisons, the stability of effects across contexts suggests that observed patterns in speech tolerance are not confined to a particular regional or institutional environment.

### Principles of speech regulations

We discussed above the debate among scholars over whether support for free speech regulations should follow a universalistic or particularistic approach. In other words, should all individuals be treated equally, or should members of historically and present-day marginalized groups receive additional protections from harmful speech? Yet we know relatively little about the public’s support for these principles (*17*), their explanatory power beyond ideology, or the extent to which they are applied consistently. To assess the role that universalistic and particularistic principles play in shaping attitudes toward restrictions on free speech, we asked respondents whether rules on potentially offensive expression should consider the identity of those targeted. Specifically, we asked:

Some believe that rules about potentially offensive speech should consider the target’s race, gender, or other characteristics as speech targeting marginalized groups can cause greater harm. Others argue against considering the target’s identity, fearing that special protections could lead to overreach and selective suppression of unpopular viewpoints. What do you support?

We refer to individuals who select the option “Rules about potentially offensive speech should consider the target’s identity (e.g., race, gender, sexual orientation, and sexual identity)” as adhering to a particularistic principle. We refer to those who selected the response option “Rules about potentially offensive speech should NOT consider the target’s identity” as following a universalistic principle. We find that about two-thirds of the students in our national sample support the particularistic principle, while a third hold universalistic views. Principle support by other demographics appears in fig. S2.

### Applying speech principles across speech severity and target identity

In an exploratory analysis, we divided the sample into two groups based on adherence to the particularistic or universalistic principle. We then examined whether this principle helped explain variation in support for speech restrictions in the scenarios that we presented in our experiments. Specifically, we estimated our preregistered models (the professor, student, and policy experiments) separately for each subsample. We highlight three key results.

**Fig. 2.**
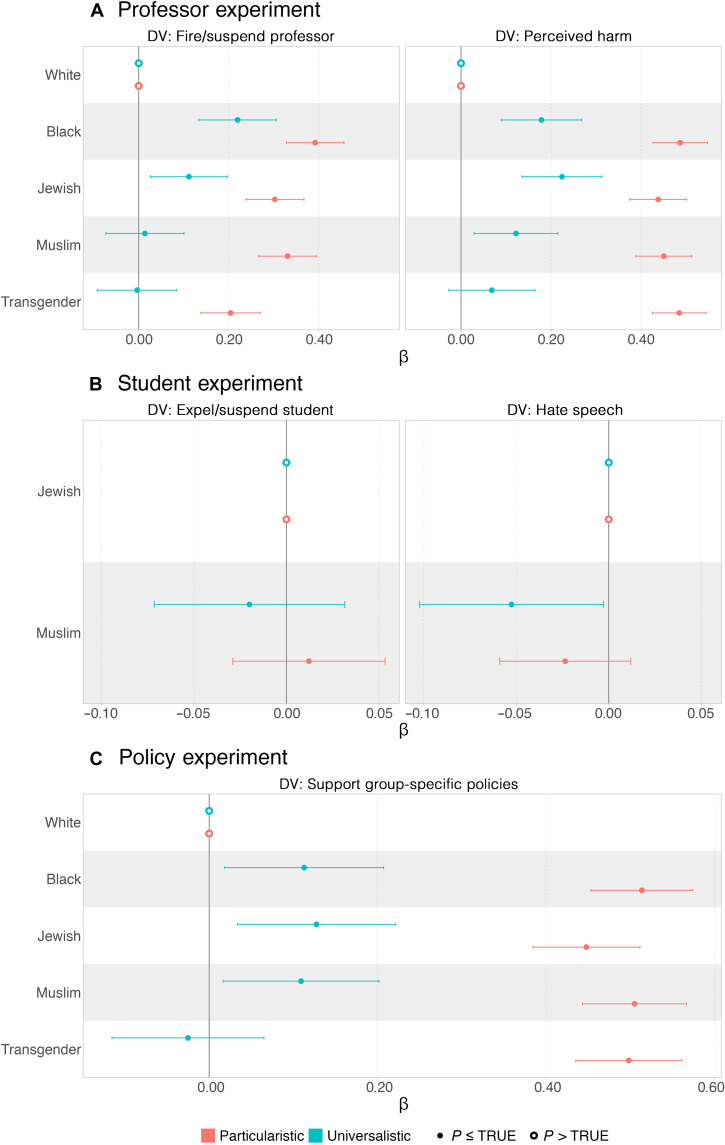
Speech principles predict support for restricting controversial speech. This figure replicates the results presented in Fig. 1, focusing on the identity of the target of speech, but subsets the sample by respondents who adhere to particularistic or universalistic principles. It shows estimated effects on support for suspending or firing a professor, suspending or expelling a student, or endorsing group-specific policies, as well as perceptions of physical or psychological harm and hate speech. (**A**) Professor experiment: left panel shows estimated effects on support for suspending or firing the professor; right panel shows estimated effects on the belief that the statement would cause physical or psychological harm. (**B**) Student experiment: left panel shows estimated effects on support for suspending or expelling the student; right panel shows estimated effects on whether the statement is classified as hate speech. (**C**) Policy experiment: estimated effects on support for endorsing a group-specific speech policy. Full regression results in tabular form shown in tables S5 to S7. DV, dependent variable.

First, students tended to follow their principles. As shown in Fig. 2A (left) and table S5, the probability of supporting suspending or firing the offending professor is close to zero and statistically insignificant for self-reported universalists when the target groups are Muslim or transgender (depicted in blue). Universalists, however, express a heightened sensitivity when the target group is Black (β = 0.22, *P* < 0.01) or Jewish (β = 0.11, *P* < 0.05), but this coefficient is substantially smaller than the estimated coefficient in the sample that include both universalists and particularists. The inclination among universalists not to punish offending professors does not simply reflect views that these statements are unlikely to cause physical or psychological damage. As shown in Fig. 2A (right), the results indicate that universalists view harmful speech to cause physical or psychological damage for Black, Jewish, and Muslim people compared to white people and, to a lesser extent, when the target is transgender people. As we show later, the universalistic principle is present even after we control for students’ political ideology and views about Israel/Palestine.

In contrast, students whom we label as particularistic (depicted in red) are more likely to support punishing professors for harmful speech when targeted against Black, Jewish, Muslim, and transgender people, compared to white individuals, and are significantly more likely to do that compared to students whom we label as universalists. It is also clear that particularists are significantly more likely to state that such statements cause physical or psychological harm when targeted against minority groups compared to white individuals. Together with our finding that particularists and universalists place equal importance on free speech (respondents were asked, “How important is protecting free speech and open expression on college campuses?” and 74 and 75% of those who identify as particularist or universalist, respectively, said “very important”), our results are consistent with the idea of a trade-off: The more groups of people are protected (in a universalistic approach), the higher is the bar one should use to restrict speech, and vice versa. In the student experiment (Fig. 2B and table S6), we find that both particularistic and universalistic students do not provide greater protections when the target group is Muslim versus Jewish people, although universalists are somewhat less likely to consider speech harmful when the target group is Muslim.

The explanatory power of the principle is also evident when we reanalyze the policy experiment (Fig. 2C and table S7). Specifically, particularists are far more likely than universalists to support group-specific speech protections for all groups, relative to white people, with estimates ranging between 15 and 18 percentage points increase. Universalists, on the other hand, were more likely to support group-specific speech restrictions for white targets compared to particularists (37% compared to 24%), but their support for group-specific speech restrictions was only modestly higher for Black, Jewish, and Muslim people (β values ranged from 0.03 to 0.13), and not different for transgender people.

One natural concern is that adherence to a particularistic or universalistic principle regarding speech restriction and protection across dominant and marginalized groups is simply an epiphenomenon of ideology. However, while conservatives are more likely to adhere to a universalistic principle and liberals to a particularistic principle (fig. S2), adherence to a principle still explains support for speech restrictions even after controlling for respondents’ ideology and other personal characteristics (Supplementary Materials, last row of tables S8 to S10).

### Deviations from principles

As mentioned, support for the principles that govern campus speech varies by various student characteristics (see fig. S2). For example, 74% of the students who identify as Democrats supported the particularistic principle, whereas 62% of the students identifying as Republicans favored the universalistic principle. Among the respondents who sympathized with Israelis during the protests, 52% endorsed the universalistic principle, while 79% of those who sympathized with Palestinians supported the particularistic principle. To further test the strength of the speech boundary principles above and beyond ideology (including position regarding the Israeli-Palestinian conflict and the Gaza war), we investigated whether adherence to universalistic and particularistic principles predicts attitudes when they conflict with ideological predispositions. This is a particularly compelling test in this context, given how the 7 October attacks and the protests against Israel’s conduct of the war in Gaza polarized campuses across the US.

**Fig. 3.**
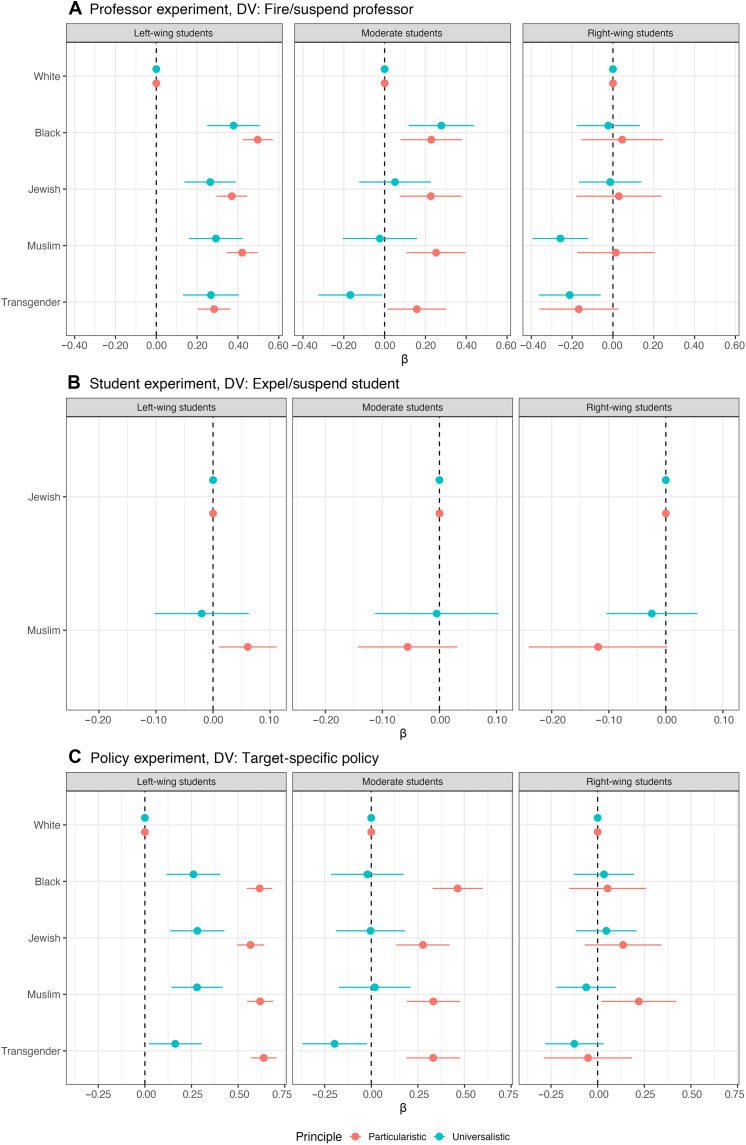
Speech principles and political ideology jointly shape support for restrictions. This figure replicates the results presented in Fig. 1, focusing on the target of speech, but subsets the sample by respondents’ adherence to particularistic and universalistic principles and their political affiliation (left-wing, moderate, or right-wing). Each panel shows the estimated effect of the target group identity on support for punitive or restrictive actions—(**A**) suspending or firing a professor, (**B**) suspending or expelling a student, and (**C**) targeting a group with policy—separately for universalists (blue) and particularists (red). Full regression results in tabular form shown in tables S11 to S19.

**Fig. 4.**
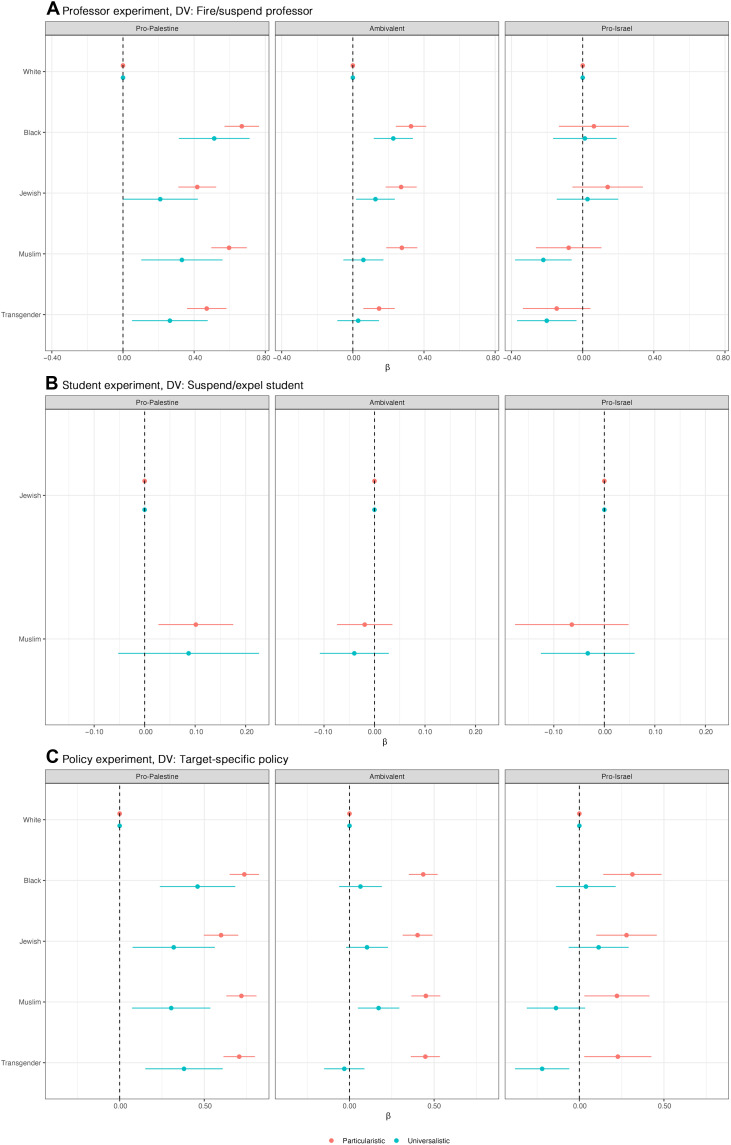
Speech principles and Israel-Palestine sympathies jointly shape support for restrictions. This figure replicates the results presented in Fig. 1 (focusing on the target of speech) but subsets the sample by respondents’ sympathies in the Israel/Palestine conflict—categorized as pro-Palestine, ambivalent, or pro-Israel—and their adherence to particularistic or universalistic principles. Each panel shows the estimated effect of the target group identity on support for punitive or restrictive actions: (**A**) suspending or firing a professor, (**B**) suspending or expelling a student, and (**C**) targeting a group with policy. Full regression results in tabular form shown in tables S20 to S28.

Specifically, we divided our sample by political ideology (left, right, or center; Fig. 3) and by a continuous sympathy index for the Israel/Palestine conflict (ranging from more pro-Palestine to more pro-Israel), splitting respondents into the bottom quartile (more pro-Palestine), the middle 50% (ambivalent), and the top quartile (more pro-Israel; Fig. 4) to evaluate whether and how political ideology or conflict-related sympathies intersect with principles regarding speech restrictions.

We find that, when other values and attitudes, such as ideology or social identity, come into play, students’ commitment to their stated principles weakens. For example, right-wing universalists express less support for suspending or firing a professor who targets Muslim and transgender individuals with hate speech compared to white students who are the target of the same statements (Fig. 3A). Left-wing students who report adherence to universalistic principles, nevertheless, demonstrate stronger support for protections for Black, Jewish, Muslim, and transgender targets compared to white students, though to a lower degree than left-wing students who report adherence to a particularistic principle.

Similar trends emerge in Fig. 4, highlighting variations in support for speech restrictions based on sentiments toward Israel and Palestine. Pro-Israel universalists, for example, are less supportive of suspending or firing a professor when the target is a Muslim or a transgender student, and pro-Palestine universalists are more likely to support suspending or firing professors who express hate toward Black or Muslim students (panel A). On the policy side, pro-Palestine universalists prefer protections for minorities over white students (panel C). We find that pro-Israel universalists and particularists are somewhat less likely to support suspending or expelling a student for engaging in repeated hate speech against Muslim targets relative to Jewish targets, but these effects are not statistically significant (panel B). Similarly, pro-Palestine universalists and particularists show no difference in their support for suspending or expelling a student for hate speech targeted at Muslim or Jewish people.

Students who are at the ideological center, or those without strong affiliations to either the Palestinian or Israeli side of the conflict (labeled as “ambivalent”), tend to uphold their free speech principles across all three experiments. For these students, adherence to the universalistic or particularistic principle has great explanatory power when analyzing the variation in how different individuals balance between freedom of speech and freedom from harm. There is one exception to this pattern in the professor experiment: Universalistic students at the ideological center are more likely to support suspending or firing a professor when the target is Black and less likely to support suspending or firing the professor when the target is transgender, but the coefficient compared to white students as targets is small and only marginally significant. While we note that ideological extremism can be correlated with unobserved factors that might affect the outcome, this finding highlights how polarization and ideology can override principled commitments to consistent standards of speech regulation (*28*).

### Robustness and generalization

Because several of our analyses were exploratory, we fielded a preregistered follow-up study with a US adult sample recruited via CloudResearch Connect. We reran the policy, professor, and student experiments using the same vignettes and outcomes. Conducted months after the student wave, this replication enables us to assess generalizability across both population and time. This sample was far more evenly divided in principle support (52% were particularistic versus 48% that were universalistic). The key patterns replicate: Perceived harm and target identity robustly predict support for restrictions, and the particularist-universalist distinction continues to structure responses, with effect signs and magnitudes closely tracking those in the student sample (see the Supplementary Materials for design and results). At the same time and as in the student wave, ideological commitments attenuate the difference between particularists and universalists. We emphasize that the student population remains substantively important in its own right: Students are central actors in campus speech controversies and the setting of institutional norms. The adult replication should be viewed as a complement that bolsters external validity, not a substitute for understanding student attitudes.

## DISCUSSION

Our study provides previously unknown evidence on how college students navigate the tension between freedom of expression and harm prevention in the context of campus speech, amid a contentious protest movement seeking to pressure college administrators to take a stance on the Gaza war. Our experimental findings show that perceptions of harm severity and the identity of the speech target strongly influence attitudes toward speech restrictions. Speech that is merely objectionable, but not perceived as especially harmful, tends to be protected. Students support greater protection from harmful speech for historically marginalized groups. We find that students generally view both Jews and Muslims as marginalized minorities at this moment in time, affording them more protection from objectionable speech compared to white students. These results do not vary by respondents’ race and ethnicity (figs. S11 to S16). Thus, the overlap in support for protecting Jewish and Muslim students likely reflects a broader orientation toward universal protection of marginalized groups rather than identical perceptions across all respondents.

Our exploratory analysis shows meaningful variation in adherence to normative principles regarding whether members of marginalized groups should be afforded greater protection from harm (“particularists”) or, instead, members of all groups should enjoy equal protection from harmful speech (“universalists”). We further show that adherence to these principles is a key factor in students’ assessment of speech restrictions. Notably, these preferences can weaken when ideological or social pressures come into play. These patterns contribute to our understanding of how individuals balance competing principles of fairness and harm prevention in polarized settings.

Our findings add to several strands of research. First, they align with studies on the physical or psychological effects of hate speech, particularly its impact on targets and observers. Prior work highlights how hateful rhetoric can cause harm, both directly and through its normalization (*29*, *30*). Our study extends this literature in two ways. First, unlike most past research, we focus on the perspectives of students on American college campuses, which have become a focal point for such debates in recent years. Consistent with prior work (*25*, *31*), we find that the severity of speech substantial influences support for restrictions, with students demonstrating stronger punitive responses to explicitly hateful statements or those that incite violence. These findings suggest that students value freedom of speech and do not restrict it lightly; they only support speech restriction when it is perceived as especially harmful. Our study further contributes to past studies on the psychological effects of hate speech in suggesting that what underlies support for greater protection for members of minority groups is that many view the same speech as more physically or psychologically harmful when the target is a member of a marginalized group as compared to a dominant group member. In other words, perceptions of harm from speech depend not only on what is said but also on who the target of that speech is.

Second, our study reinforces prior research showing that the target’s identity influences support for speech restrictions (*12*, *32*). Like earlier work, we find that speech targeting minority or marginalized groups is perceived as more harmful and elicits greater support for sanctions, consistent with norms favoring the protection of lower-status groups (*27*). However, our contribution lies in systematically comparing responses across a wide range of identity groups—including Black, Jewish, Muslim, white, and transgender individuals—and isolating these effects using experimental manipulations. The study’s timing, amid heightened tensions on college campuses due to the Gaza protests, offers important insights into how polarized contexts influence attitudes toward speech directed at Jewish and Muslim communities. Focusing on college students, active participants, and observers in these debates, our findings extend the literature to a population and setting where questions of free speech and identity are especially fraught.

Our exploratory analysis of particularistic and universalistic principles offers further insights into the possible mechanisms shaping these attitudes. While most students favor protecting marginalized groups, their commitment to these principles varies somewhat depending on ideological alignment and other social factors. This finding complements work in political behavior, showing how partisan loyalties can influence the application of abstract principles (*28*). At the same time, we find that students at the ideological center are more consistent in their adherence to free speech principles, echoing research suggesting that those without strong partisan attachments may be better positioned to apply normative frameworks uniformly (*33*). The 7 October attacks and the subsequent war provide an important contextual backdrop for interpreting these patterns. These events heightened the salience of in-group and out-group identities, potentially magnifying ideological pressures and making it more difficult for students to maintain their stated principles. This context may help explain why deviations from principles were particularly pronounced among students with strong stances regarding the Israeli-Palestinian conflict as moments of heightened identity salience often bring underlying biases and social pressures to the fore.

Together, our findings contribute to ongoing debates about the boundaries of free speech on college campuses, particularly in contexts of heightened polarization and identity-based tensions. The patterns we uncover shed light on the underlying factors that fuel disagreements over campus speech, including the competing normative commitments to universal protections versus targeted safeguards for marginalized groups. As campuses serve as microcosms for broader societal debates, understanding these dynamics offers insights into the challenges of fostering inclusive dialogue while navigating the limits of free expression. Future research can build on these findings to explore how such disagreements evolve in response to changing social and political contexts.

## MATERIALS AND METHODS

### Overview

We conducted a nationally representative survey of US college students to study how institutional roles and conflict-related cues shape support for restricting controversial speech. The design combines three complementary survey experiments—a policy experiment, a professor vignette experiment, and a student vignette experiment—along with measures of political attitudes and conflict-related perceptions. Descriptive characteristics of the sample are reported in table S1 and fig. S1.

### Ethics approval

The project has been approved by the Institutional Review Boards of the University of Pennsylvania (protocol no. 856042), the Columbia University (protocol no. AAAV2719), University of Colorado (APP001-1), and the Stanford University (protocol no. 75765).

### Sample and fieldwork

We partnered with College Pulse, an online survey company specializing in researching American college students’ attitudes, preferences, and behaviors. College Pulse maintains a panel of more than 800,000 undergraduate respondents from more than 1500 2- and 4-year colleges and universities across all 50 states.

Fieldwork took place between 6 June 2024 and 9 July 2024. The data are based on a nationally representative sample of 3065 full-time undergraduate students enrolled in 4-year colleges and universities across the US.

Respondents were selected from College Pulse’s American College Student Panel, a diverse panel of more than 800,000 verified college students and recent graduates from more than 1500 2- and 4-year institutions across all 50 states. Panelists were recruited through multiple strategies—such as digital advertising, opt-in email campaigns, and collaborations with student organizations affiliated with universities—to ensure broad representation. The panel reflects the diversity of the US college population, including students from large public universities, small private colleges, online institutions, historically Black colleges and universities (e.g., Howard University), women’s colleges (e.g., Smith College), and religiously affiliated institutions (e.g., Brigham Young University).

To confirm enrollment, College Pulse uses a two-step verification process. All panelists must provide a valid “.edu” email address to join, and survey respondents had to affirm their current full-time enrollment in a 4-year degree program. Survey invitations were sent directly to students’ .edu email addresses or via push notifications in the College Pulse mobile app, available on both iOS and Android.

### Experimental design and procedure

Each respondent completed all three experiments. Treatment assignment within each experiment was randomized. The order of the three experiments within the survey was held fixed; because treatment assignment is randomized within each experiment, the internal validity of each experiment’s causal estimates is preserved.

In the policy experiment, we asked respondents whether they would favor or oppose a rule making it a violation of campus policies to say offensive things in public against different target groups, which were randomized to include either Black, Jewish, Muslim, transgender, or white people. The measure was dichotomized so that those who strongly or somewhat supported such a policy were coded as one, and zero otherwise.

In the professor experiment, respondents read a single scenario, in which we randomized the identity of the targeted group the professor insults (either Black, Jewish, Muslim, transgender, or white people), the context of the objectionable statement (either a campus protest, a class lecture, or a private text message), and the severity of the speech (two levels: “play the victim to get special treatment” and “the US will be a better place without [target group]”). The text of the vignette is as follows:

Imagine that in [context], a professor from your college expressed their opinion that [target group] [speech severity].

After reading the (randomized) scenario, we asked respondents to evaluate the likelihood that the statement would cause physical or psychological harm to the targets (we dichotomized responses such that harm is coded as one if respondents viewed the speech as likely or very likely to cause harm, and zero otherwise) and to share their views on how the university should respond. Response options included taking no action, publicly condemning the behavior without punishment, punishing the professor without suspension, suspending the professor for a fixed term, and firing the professor (we dichotomized responses such that the recommended university response is coded as one if respondents supported firing or suspending the professor and zero otherwise).

In the student experiment, respondents were asked to read a hypothetical statement repeatedly made by a student in their university, even after being informed multiple times that some might find it offensive. The scenario focused on two target groups: Jews and Muslims. Five objectionable statements were referencing Jews, and five statements referencing Muslims, capturing differences in speech severity/harmfulness. These 10 statements were randomized so that each respondent read only one:

1) Most [Jews/Muslims] in America are more loyal to [Israel/Islam] than to America.

2) Most [Jews/Muslims] use shady practices to get what they want.

3) Most [Jews are Zionists/Muslims are Jihadists] who promote an oppressive ideology.

4) The world would be better off with fewer [Jews/Muslims].

5) [Jews/Muslims] are the root of all evil, and we need to deal with them by any means necessary.

The professor and student experiments were designed to reflect realistic differences in speech contexts and institutional hierarchies. In the student experiment, the warning that a statement might be found offensive was included to mirror pedagogical norms in which students are cautioned about acceptable boundaries of speech. In the professor experiment, we omitted such warnings, assuming that faculty are already aware of these norms and are held to higher standards of accountability. These differences reflect distinct power dynamics and institutional expectations; thus, the two experiments were intended as complementary tests.

Our design did not seek to equate Zionism with Jihad, nor does it require respondents to interpret these terms as analogous. The manipulation randomized exposure to derogatory messages targeting Jews (via Zionism) or Muslims (via Jihad), using parallel phrasing to approximate similar levels of offensiveness. We did not define these terms for respondents, as our interest lies in how participants themselves interpret and react to such language in a realistic context. While Jihad was likely perceived as more derogatory than Zionism, the fact that patterns mirror those in the professor experiment, where objectionable speech was held constant, suggests our findings reflect sensitivity to group-based targeting rather than equivalence between the two terms.

### Additional materials

In addition to the hypothetical experiments described above, we also presented respondents with a third scenario that included real statements made by university professors that some might find harmful. Examples included a professor celebrating the death of Barbara Bush on social media, calling a Diversity, Equity, and Inclusion (DEI) training “anti-intellectual and totalitarian,” posting “All I want for Christmas is white genocide,” and stating in class that they would “kill” Colin Kaepernick when asked about his protest during the national anthem. For space constraints, we do not include this experiment here, but full descriptions of the statements and the results can be found in the Supplementary Materials.

### Measures

After reading the (randomized) scenario, we asked respondents whether they would classify the statement as hate speech and share their views on how the university should respond. Response options included taking no action, publicly condemning the behavior without punishment, punishing the student without suspension, suspending the student for a fixed term, and expelling them (we dichotomized responses such that recommended university response is coded as one if respondents supported expelling or suspending the student and zero otherwise). In both the professor and student experiments, we assume that support for severe punishment (suspension/firing and suspension/expelling) represent implicit support for speech restrictions and low tolerance for protecting harmful speech.

Last, we asked respondents about their views on the Israeli-Palestinian conflict, including their reactions to the 7 October attacks, their sympathy toward both Israeli and Palestinian people, their support for or participation in campus protests (including disruptive tactics like encampments), their opinions on the university administration’s response to the protests, and their stance on various speech policies, such as inviting controversial speakers to campus and maintaining university neutrality. We use some of these measures to test whether students follow their particularistic or universalistic principles, even when the targeted group is one toward which they are personally antagonistic. The Supplementary Materials provides more details on these additional questions.

### Preregistration and ethics

We preregistered our hypotheses and planned analyses before fielding the survey, at the Open Science Framework. The study was reviewed and approved by the Institutional Review Boards of the University of Pennsylvania, the Columbia University, the University of Colorado, and the Stanford University. As is the convention, we solicited informed consent at the beginning of the survey, as a condition for proceeding through it.

To estimate the effect of the treatments on the outcomes, we used OLS regression models for each experiment. The models are specified as follows:

1) Professor experiment

$$\begin{array}{c} Y_{i}=\beta_{0}+\beta_{1}{\text{TargetGroup}}_{i}+\beta_{2}{\text{SpeechSeverity}}_{i}\\ +\;\beta_{3}{\text{Context}}_{i}+X_{i}\gamma +\epsilon_{i} \end{array}$$

2) Student experiment

$$\begin{array}{c} Y_{i}\;=\;\beta_{0}\;+\;\beta_{1}{\text{SpeechSeverity}}_{i}\;+\;\beta_{2}{\text{StatementSeverity}}_{i}\\ +\;X_{i}\gamma \;+\;\epsilon_{i} \end{array}$$

3) Policy experiment

$$Y_{i}\;=\;\beta_{0}\;+\;\beta_{1}{\text{RandomTargetGroup}}_{i}\;+\;X_{i}\gamma \;+\;\epsilon_{i}$$

For all three models, $Y_{i}$ represents the outcome variable, which varies by experiment. In the professor experiment, this includes respondents’ support for punitive actions against the professor or perceptions of physical or psychological harm caused by the speech. In the student experiment, it measures support for disciplinary actions against a student making the statements and the perception of the statement as hate speech. In the policy experiment, it captures support for a policy banning offensive speech targeting specific groups. The variable ${\text{TargetGroup}}_{i}$ captures the effect of the identity of the group targeted by the speech (e.g., Black, Jewish, Muslim, transgender, or white individuals, which we treat as the reference category). The variable ${\text{SpeechSeverity}}_{i}$ (in the professor experiment and student experiment) accounts for the severity of the speech, distinguishing between less harmful and explicitly hateful statements. ${\text{Context}}_{i}$ (in the professor experiment) represents the effect of the context in which the speech occurred (e.g., during a protest, in a classroom, or in private). We report all regression models with and without a vector of control variables ($X_{i}$), which includes respondents’ gender, race, sexual orientation, ideology (liberal-conservative seven-point scale), and their sympathies toward Israelis and Palestinians. Last, $\epsilon_{i}$ is the error term.
