# Supplementary material for: Expression at the edge: Free speech boundaries amidst the Gaza crisis
Source: Sci Adv. 2026 Apr 15;12(16):eaea5427. doi: 10.1126/sciadv.aea5427 (PMC13082313; doi:10.1126/sciadv.aea5427)
Supplement: Supplementary file 1 — Supplementary Text Figs. S1 to S26 Tables S1 to S44 [file sciadv.aea5427_sm.pdf]

Science Advances
AAAS

Supplementary Materials for

Expression at the edge: Free speech boundaries amidst the Gaza crisis

Ran Abramitzky *et al.*

Corresponding author: Yphtach Lelkes, ylelkes@upenn.edu

Sci. Adv. **12**, eaea5427 (2026)
DOI: 10.1126/sciadv.aea5427

This PDF file includes:

Supplementary Text
Figs. S1 to S26
Tables S1 to S44

Information about the sample

Table S1 provides the distribution of the demographics of the college students in our main experiment. Figure S2 shows differences in universalistic versus particularistic principle support by demographics. Figure S1 shows the geographic distribution of our respondents.

Wording for Experiments

The full questionnaire that includes all variables that were collected appears on the OSF page: https://osf.io/85hfq/

Professor Experiment

Stimuli Imagine that in [context], a professor from your college expressed their opinion that [group][play the victim to get special treatment OR the U.S. will be a better place without] [group].

Context: campus protest, a class, or a private text message

Target group identity: Black/Jewish/white/Muslim/Transgender

Speech severity: play the victim to get special treatment/the U.S. will be a better place without

Follow-up Questions How likely is it that the professor's statement causes harm? (Very unlikely / Unlikely / Neutral / Likely / Very likely)

How should the university respond? (Do nothing/Publicly condemn but refrain from punishment/Punish (without suspension)/Suspend without pay for a fixed term/Fire)

Student Experiment

Stimuli [Randomized to one statement and one target group]

Most [Jews/Muslims] in America are more loyal to [Israel/Islam] than to the USA.

Most [Jews/Muslims] use shady practices to get what they want.

Most [Jews are Zionists/Muslims are Jihadists] promote an oppressive ideology.

The world would be better off with fewer [Jews/Muslims].

[Jews/Muslims] are the root of all evil and must be dealt with by any means necessary.

Follow-up Questions Is this sentence hate speech? Options: Not offensive/ Offensive but not hate speech/ Yes, hate speech

How should the university respond to a repeat offender? Options: Do nothing/Publicly condemn/Punish (without suspension)/Suspend without pay/Expel

The Policy Experiment

Would you favor or oppose a rule making it a violation of campus policies to say offensive things in public against [Black/Jewish/white/Muslim/Transgender] people? (Strongly support / Support / Neither oppose nor support / Oppose / Strongly oppose)

Additional Survey Questions

1. Many people have taken sides in the current war between Israel and Hamas. Which group do you sympathize with more strongly in the context of the Israeli-Palestinian conflict?

    - Israeli people
    - Palestinian people
    - Both equally
    - Neither
    - I prefer not to answer

2. On October 7, 2023, Hamas brigades invaded Israel, killing over 1,100 Israeli citizens, including elderly people, women, babies, and unarmed men. Please give your opinion on the following two statements:

    Statement A: October 7 was a horrific terror attack that cannot, and should not, be justified under any circumstances.
    Statement B: October 7 was a legitimate act of armed resistance to decades of Israeli occupation.

    Do you agree more with statement A or statement B?

    - A
    - B
    - Neither
    - Don't know

3. Now think about October 6, 2023, a day before Hamas's terror attack on Israel. At that time, which group did you sympathize with more strongly?
    - Israeli people
    - Palestinian people
    - Both equally
    - Neither
    - I prefer not to answer
4. On a scale from 0 to 10, where 0 represents the most pro-Israel position and 10 represents the most pro-Palestinian position, how would you rate the position of the following individuals/groups?
    - Yourself
    - Most students on your campus
    - The university administration/leadership
    - Most professors on your campus
5. On a scale from 0 to 10, where 0 represents the most pro-Palestinian position and 10 represents the most pro-Israel position, how would you rate the position of the following individuals/groups?
    - Yourself
    - Most students on your campus
    - The university administration/leadership
    - Most professors on your campus
6. How important is protecting free speech and open expression on college campuses?
    - Very important
    - Somewhat important
    - Not very important
    - Not important at all

Additional Pre-Registered Experiments

This section includes results from three other experiments that appeared in the survey instrument.

Real Statements Experiment

In order to introduce some more realism to the survey, we presented respondents with one of four true stories we have culled from running online search. Two of the stories are objectionable but not necessarily harmful (1-2), and two stories describe speech that is arguably hateful (3-4). Respondents read the following question: *Below is a real story of a university professor who made comments that some might find objectionable and harmful.*

1. A professor posted on social media after the death of the former First Lady Barbara Bush, the wife of President George W. H. Bush, ”*I'm happy the witch is dead. Can't wait for the rest of her family to fall to their demise the way 1.5 million Iraqis have. Byyyeeeeeeee.”*

2. A university sent a mass email encouraging all faculty members to participate in Diversity, Equity, and Inclusion (DEI) training. A professor replied, mocking the DEI event and discouraging participation, adding, *"The event will be anti-intellectual and totalitarian: there'll be bromides, clichés, and amen-corner rah-rahs in plenty."*

3. Professor posted on social media, *"All I want for Christmas is white genocide."*

4. A professor was asked in class about their view on Colin Kaepernick, the NFL quarterback who knelt during the national anthem to protest police violence against Black people. Their response was, *"I would kill him".*

World Events Experiment

Do you believe that your university should publish official statements in response to significant world events, such as [randomize: George Floyd's murder; Hamas's attack on Israel on October 7; Israel's invasion of Rafah; the 2016 gay nightclub shooting; the Russia-Ukraine conflict], or should it avoid taking an official position?

- The university should publish official statements

- The university should not issue official statements
- Don't know

The results appear in Table ??.

Campus events

Some students believe that controversial speakers should not be allowed to speak on campus since inviting them to campus threatens inclusivity and legitimizes their position. Others believe that academic freedom means allowing even objectionable voices to be heard and that banning speech on campus runs the danger of a slippery slope.

Now, assume the following has been invited to speak at a campus event. Do you think the university should intervene and cancel the event with [randomize: Benjamin Netanyahu, Prime Minister of Israel and leader of the right party "Likud"; Enrique Tarrio, leader of Proud Boys, a far-right group; George Soros, a Businessman and philanthropist for liberal causes; Ismail Haniyeh, the leader of Hamas's political arm; Itamar Ben-Gvir, Israel's National Security Minister and leader of the far-right party "Jewish Power"; Mahmoud Abbas, the President of the Palestinian Authority; Rupert Murdoch, Media executive (Fox News) and philanthropist for conservative causes; Samuel Miller, an activist with Antifa, a far left group]

Results from this experiment appear in Tables ?? and ??

Subgroup analyses

We examine whether the results differ by respondent race (white non-Hispanic, Black, Hispanic, Asian, other), attitudes toward Israel and Palestine, and the the partisanship of the respondent's states. These results appear in Figure S9 through Figure S22.

Adult Replication (CloudResearch Connect): Design, Measures, and Results

To complement the (partly) exploratory analyses in the student wave, we fielded a follow-up experiment among U.S. adults (N=2000) recruited via CloudResearch Connect in November 2025. We re-ran the *policy*, *professor*, and *student* experiments using the same vignettes and primary outcomes, allowing us to assess generalizability across both populations and time. The pre-registration appears in the OSF directory.

Measures and harmonization. In addition to the main outcomes reported in the student wave, the replication added two harmonized outcomes to *both* the professor and student experiments so that *all* respondents were asked: (i) whether the statement constitutes hate speech, and (ii) whether the statement is likely to cause physical or psychological harm. This ensures comparable "hate" and "harm" measures across all conditions and targets.

Results summary. The results for the adult replication appear in Figures S23:S26 and on the OSF page. The adult replication reproduces the core findings from the student sample and closely matches the size of the main effects. For example, in the professor experiment, harmful speech about Black people increases support for firing the professor by 0.33 in the student sample and 0.21 in the adult sample, with similarly close correspondence for Jewish and Muslim targets. In the policy experiment, target-group effects on support for banning offensive statements are larger in the student data than in the adult sample (≈ 0.30–0.38 vs. 0.14–0.20 among adults), and the pattern of greater protection for minority targets than for White targets is identical across samples. When we stratify by speech principles, ideology, and sympathies, the direction and structure of the interactions are the same in both studies; some subgroup contrasts are modestly smaller and less precisely estimated in the adult data, but we do not observe any sign reversals or qualitatively new patterns. The main difference between samples lies in the distribution of principles: students are more likely to endorse particularistic protections, whereas adults are roughly evenly divided between particularists and universalists.

Complete materials and outputs. All replication materials—including stimuli, code, machine-readable tables, and de-identified data—are available on our OSF project page: Open Science Framework.

Notes on analysis. Unless otherwise noted, models, coding, and specifications follow those described in the Materials and Methods for the student wave; any deviations are documented in the OSF analysis scripts.

Complete materials and outputs. All replication materials—including stimuli, code, machine-readable tables, and de-identified data—are available on our OSF project page: Open Science Framework.

Notes on analysis. Unless otherwise noted, models, coding, and specifications follow those described in the Materials and Methods for the student wave; any deviations are documented in the OSF analysis scripts.

Supplementary Figures

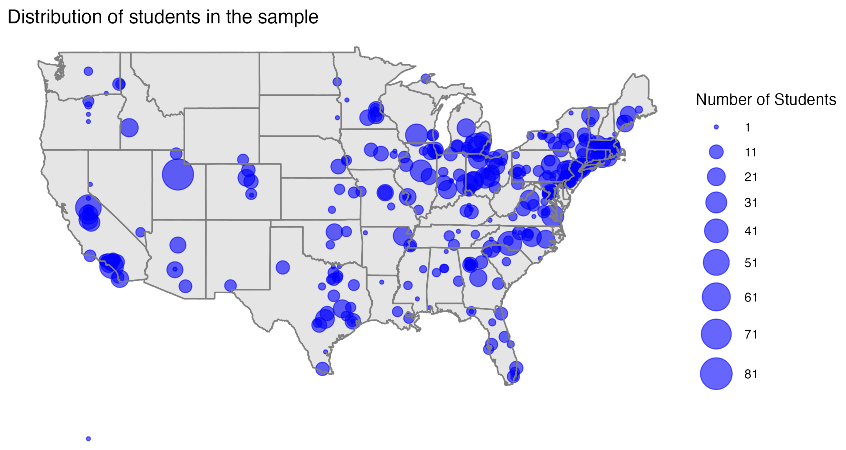

Figure S1: **Geographic distribution of survey respondents.**

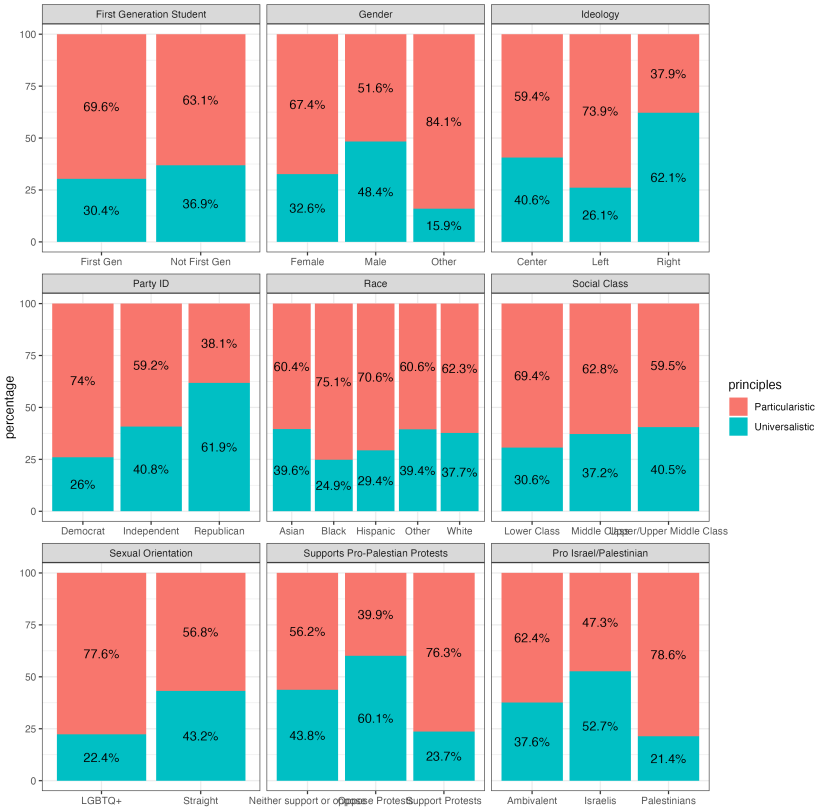

Figure S2: **Demographic differences in support for universalistic versus particularistic principles.**

Student Views on Speech Restrictions by Target and Severity Mean Values

These figures shows the predicted outcomes from Figures 1-4 in the main text.

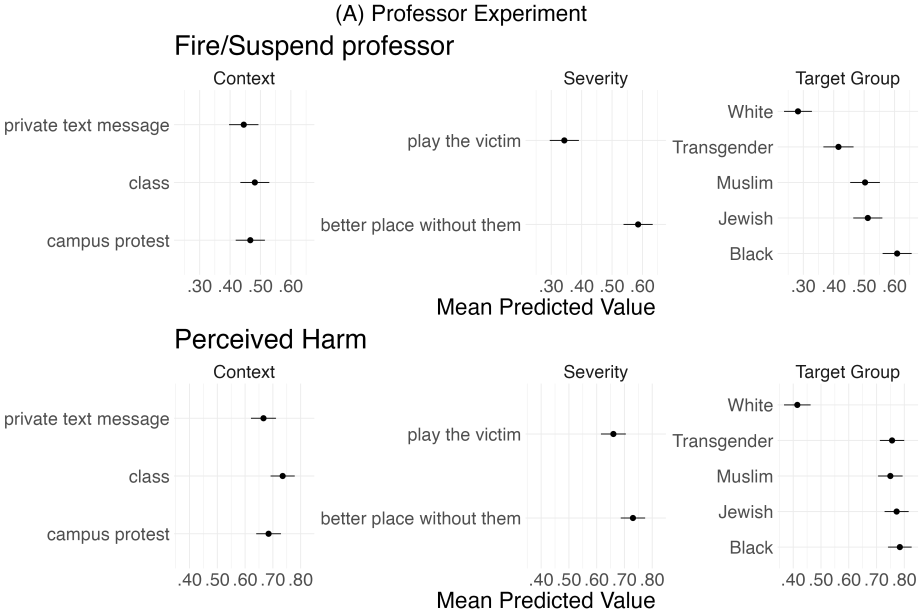

Figure S3: **Mean predicted probabilities for professor experiment outcomes by target group, severity, and context.**

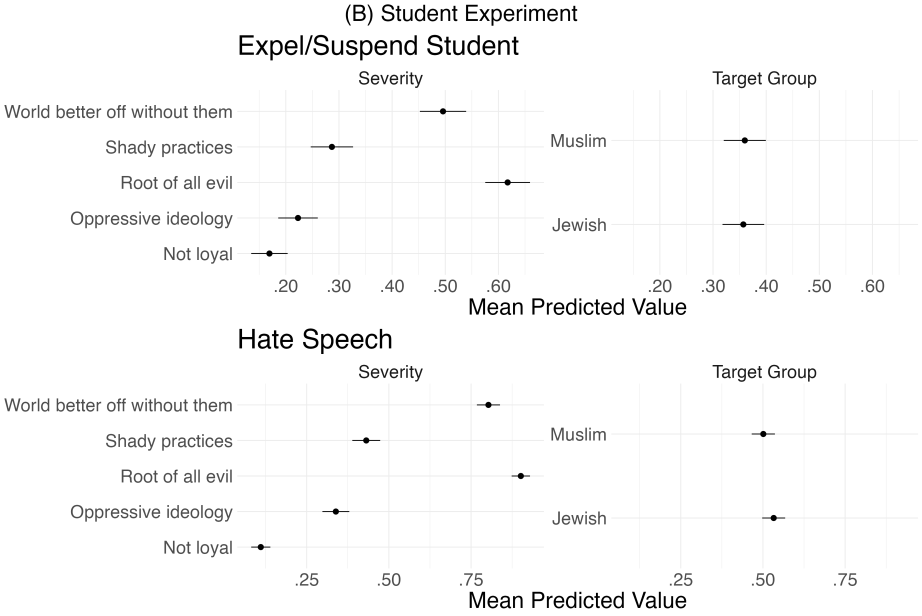

Figure S4: **Mean predicted probabilities for student experiment outcomes by target group and statement severity.**

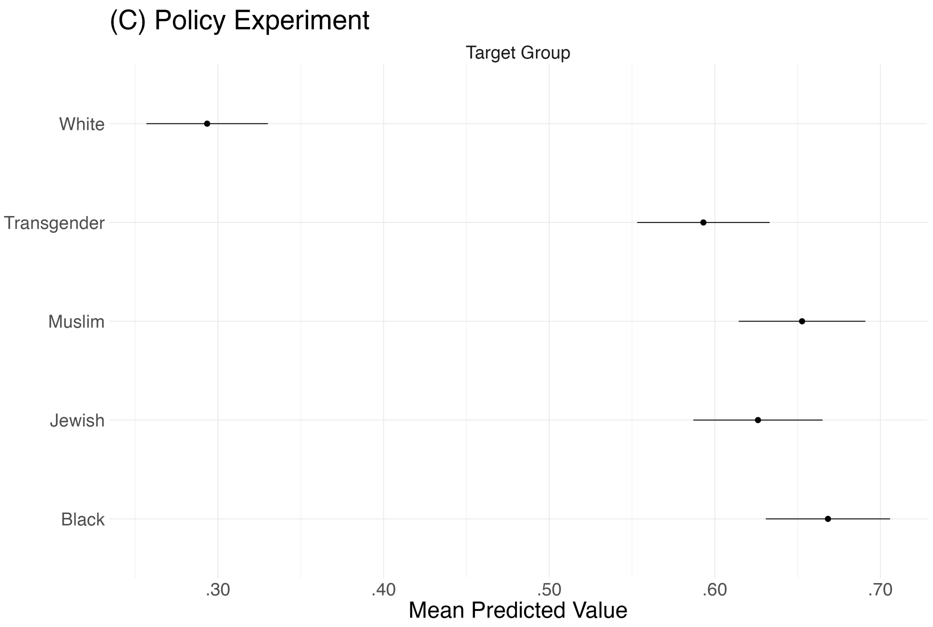

Figure S5: **Mean predicted probabilities for policy experiment outcomes by target group.**

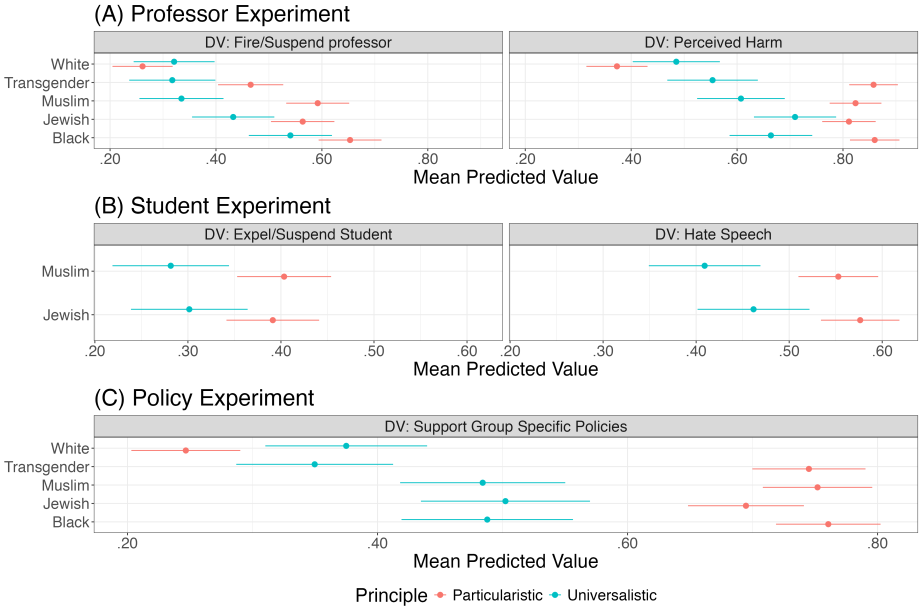

Figure S6: **Mean predicted probabilities by universalistic versus particularistic principles.**

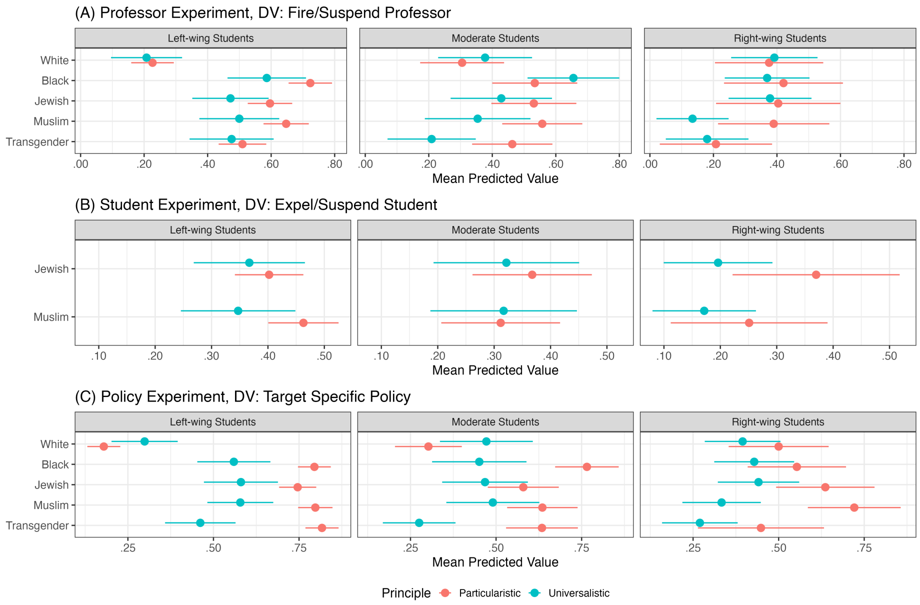

Figure S7: **Mean predicted probabilities showing interaction effects between experimental factors.**

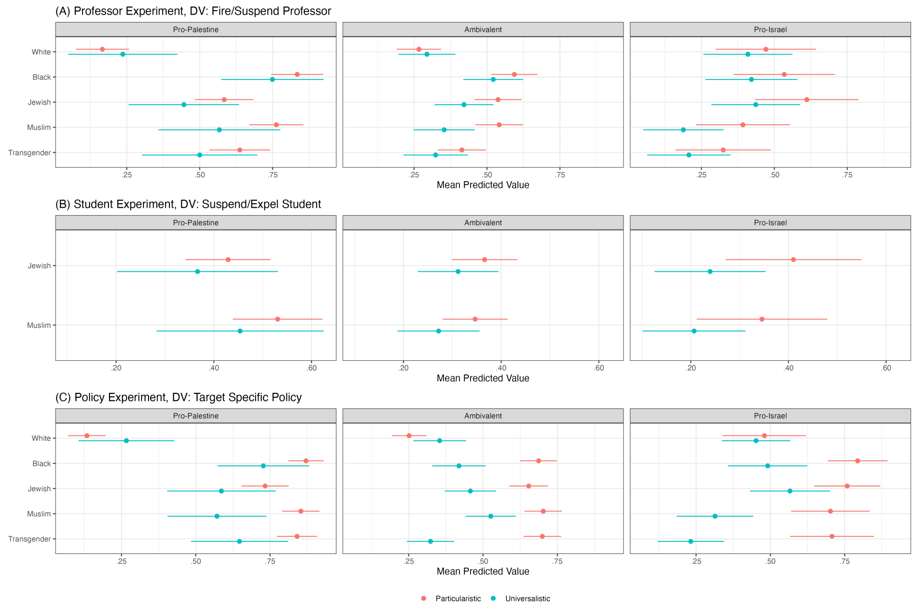

Figure S8: **Mean predicted probabilities for deviations from baseline experimental conditions.**

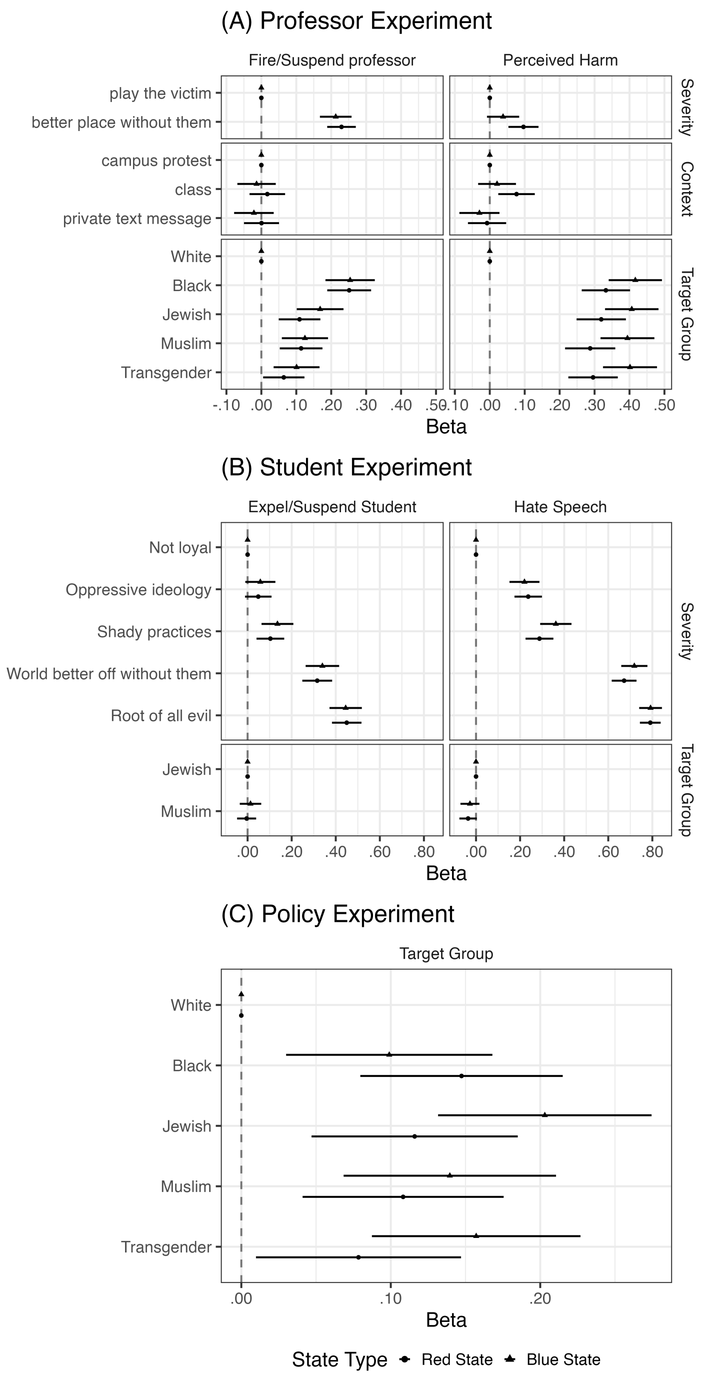

Figure S9: **Professor and student experiment results by state partisanship.**

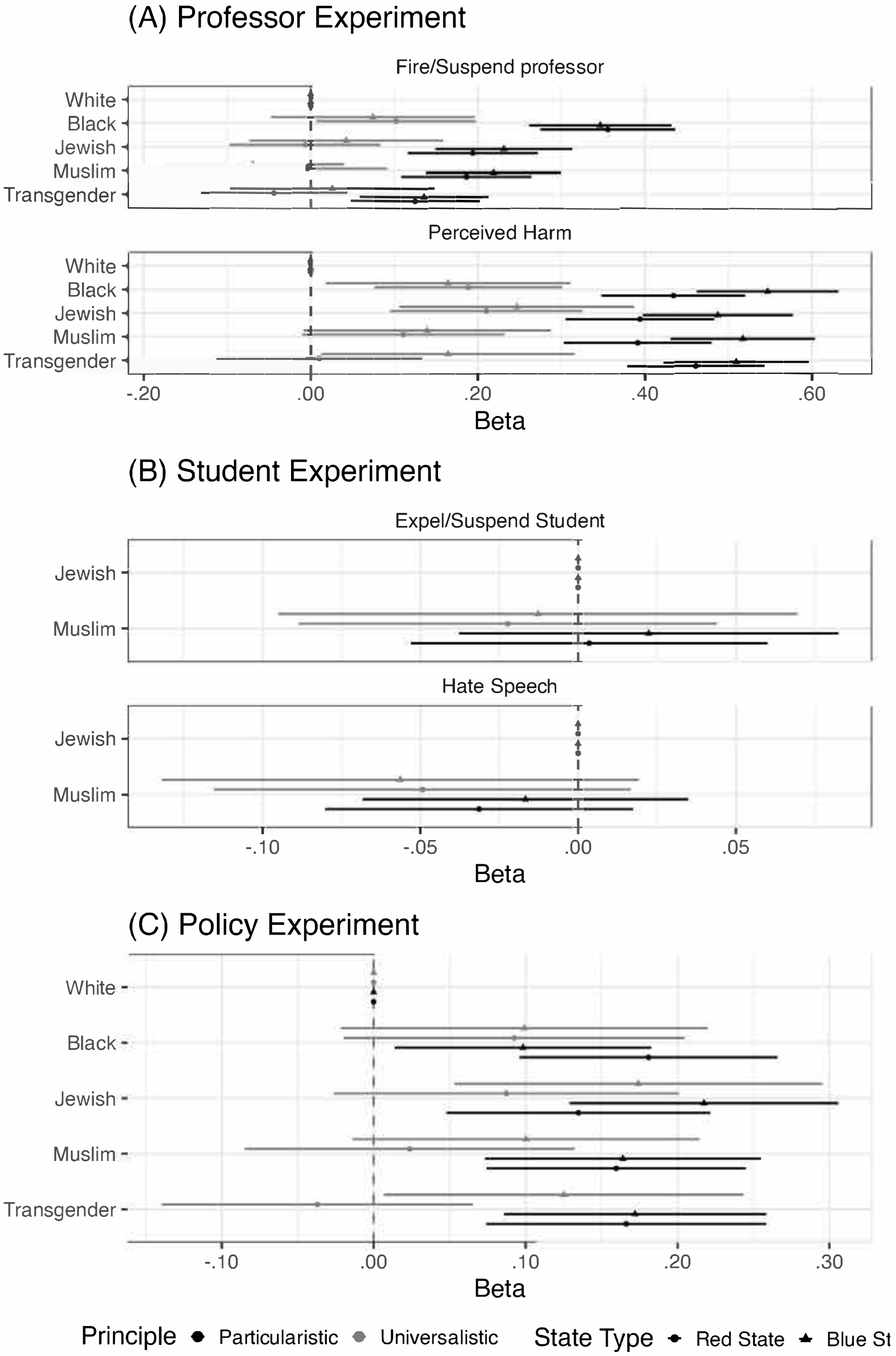

Figure S10: **Principle-based results by state partisanship.**

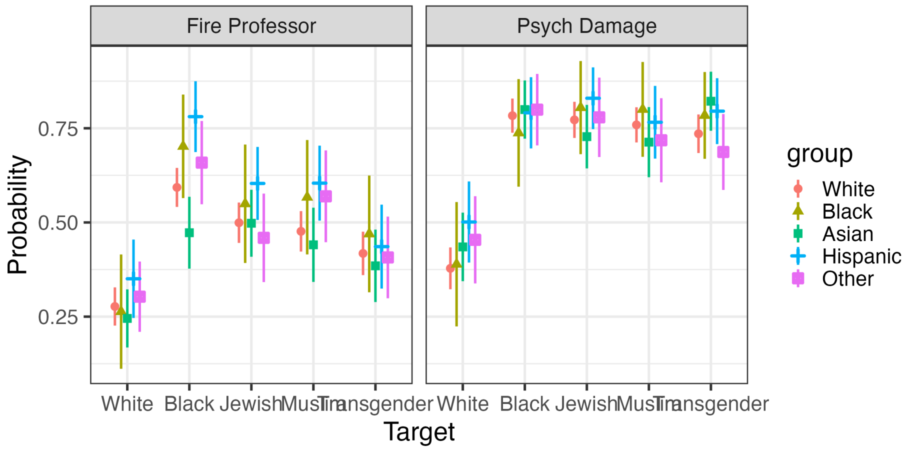

Figure S11: **Predicted probabilities from professor experiment models by respondent race.**

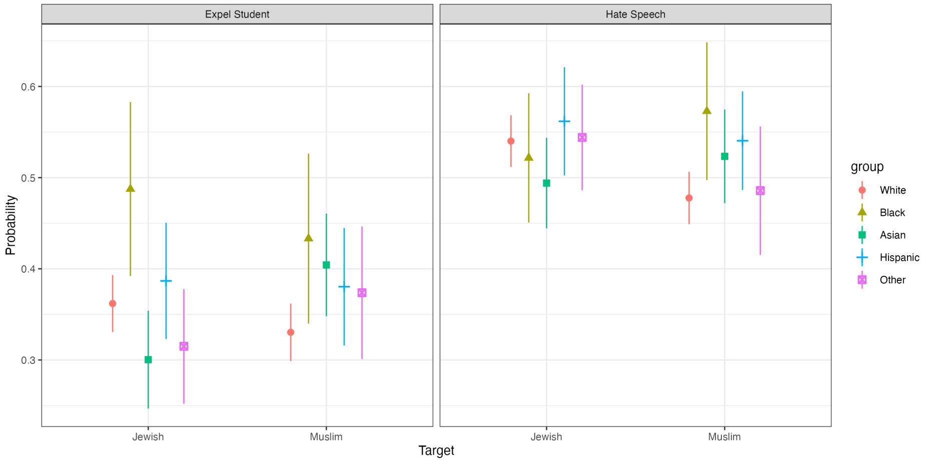

Figure S12: **Predicted probabilities from student experiment models by respondent race.**

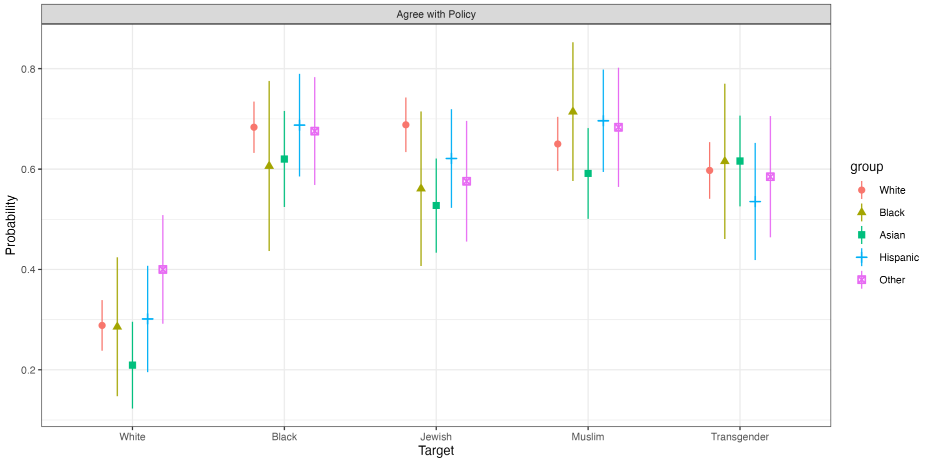

Figure S13: **Predicted probabilities from policy experiment models by respondent race.**

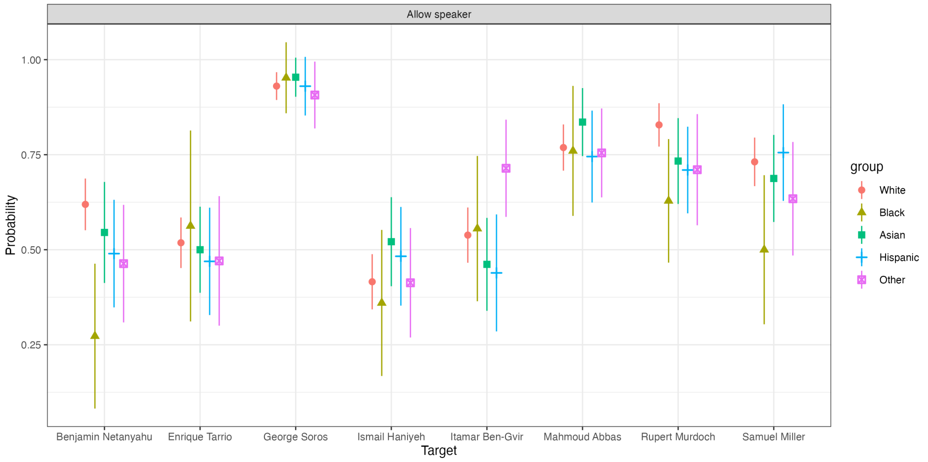

Figure S14: **Predicted probabilities from speaker event experiment models by respondent race.**

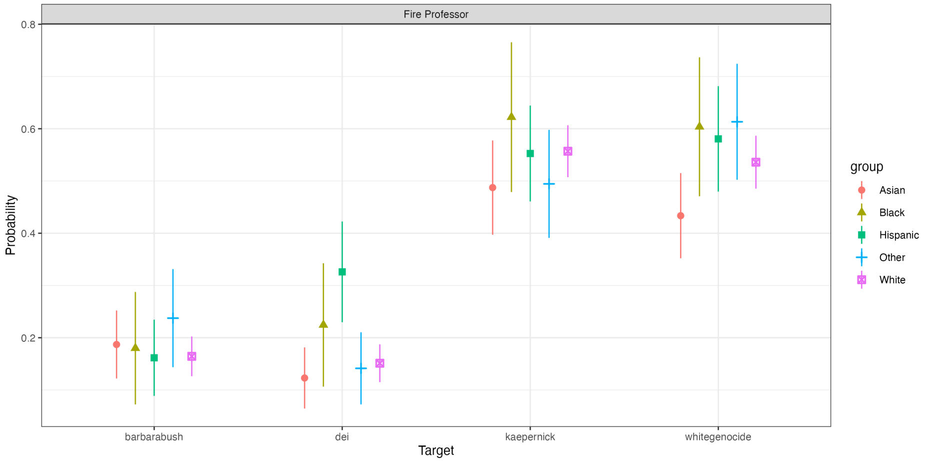

Figure S15: **Predicted probabilities from real professor statement experiment models by respondent race.**

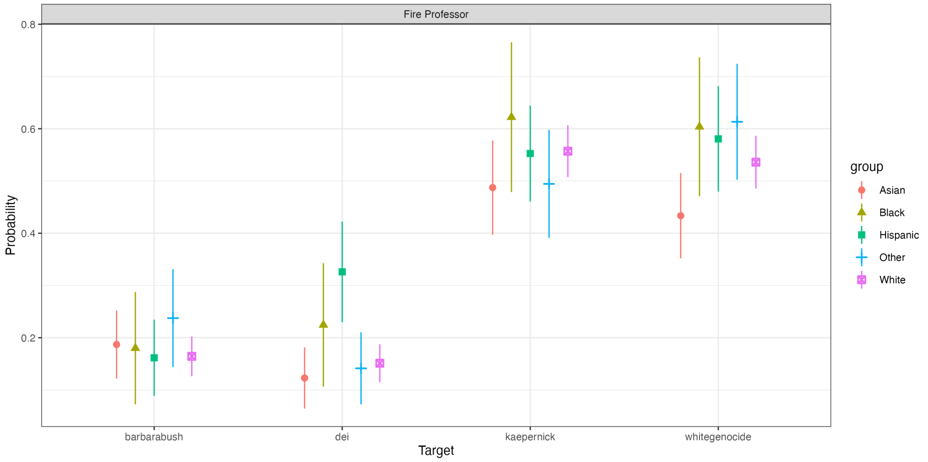

Figure S16: **Predicted probabilities from world events experiment models by respondent race.**

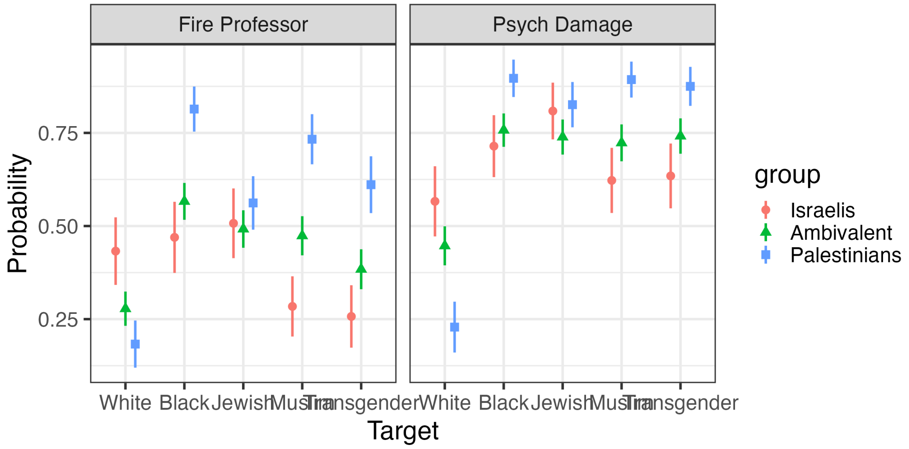

Figure S17: **Predicted probabilities from professor experiment models by respondent Israel-Palestine sympathy.**

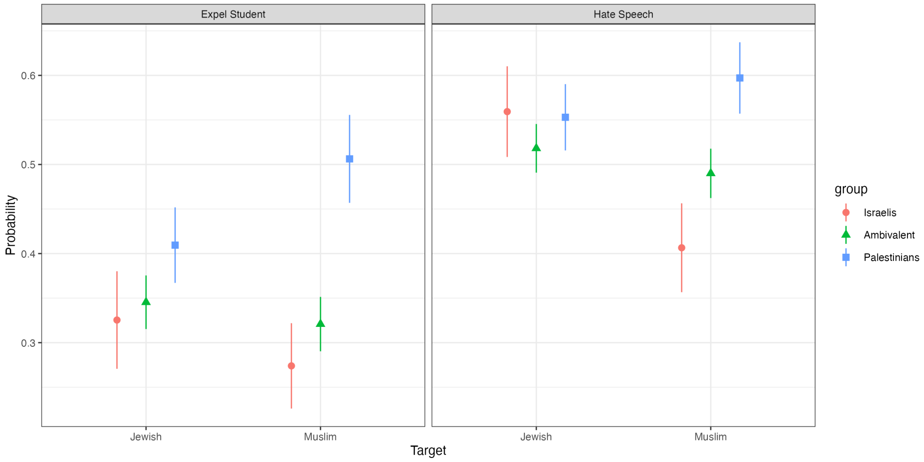

Figure S18: **Predicted probabilities from student experiment models by respondent Israel-Palestine sympathy.**

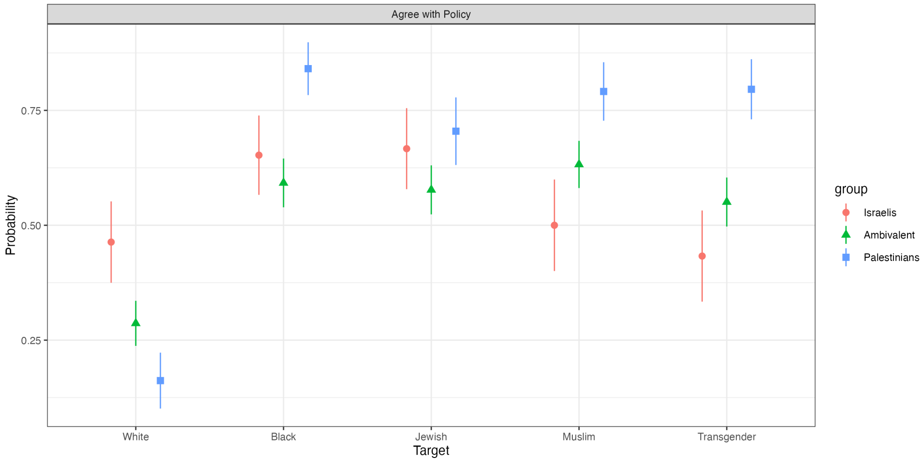

Figure S19: **Predicted probabilities from policy experiment models by respondent Israel-Palestine sympathy.**

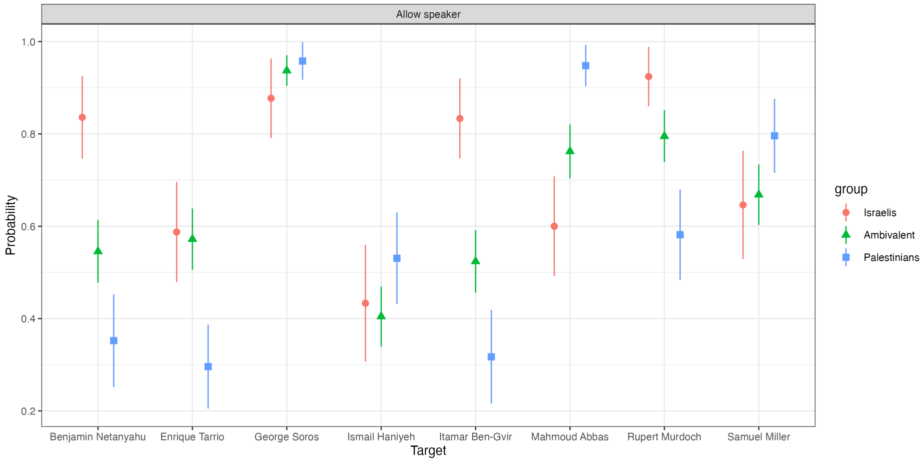

Figure S20: **Predicted probabilities from speaker event experiment models by respondent Israel-Palestine sympathy.**

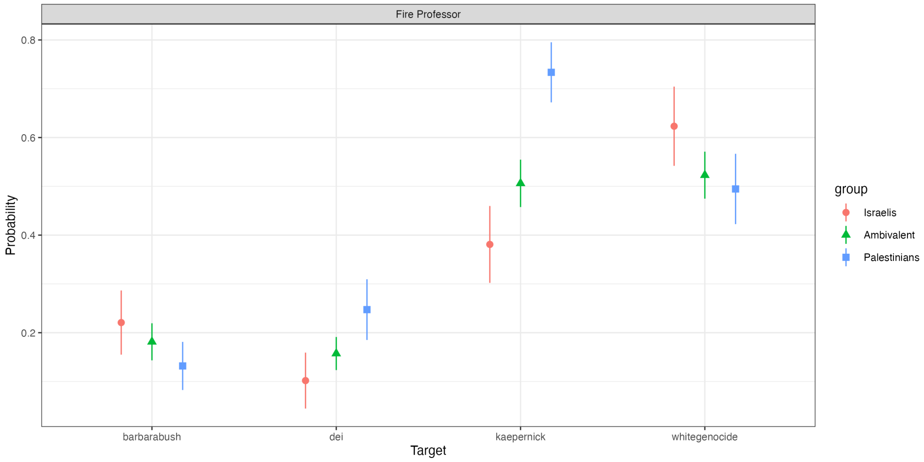

Figure S21: **Predicted probabilities from real professor statement experiment models by respondent Israel-Palestine sympathy.**

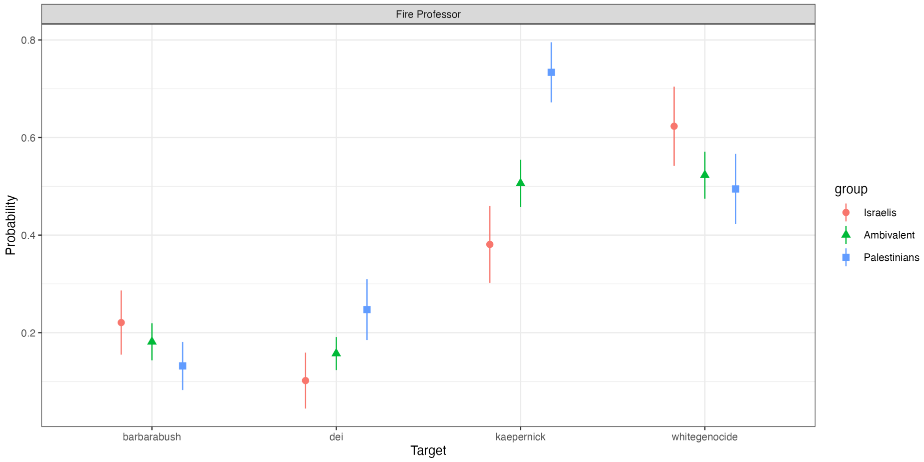

Figure S22: **Predicted probabilities from world events experiment models by respondent Israel-Palestine sympathy.**

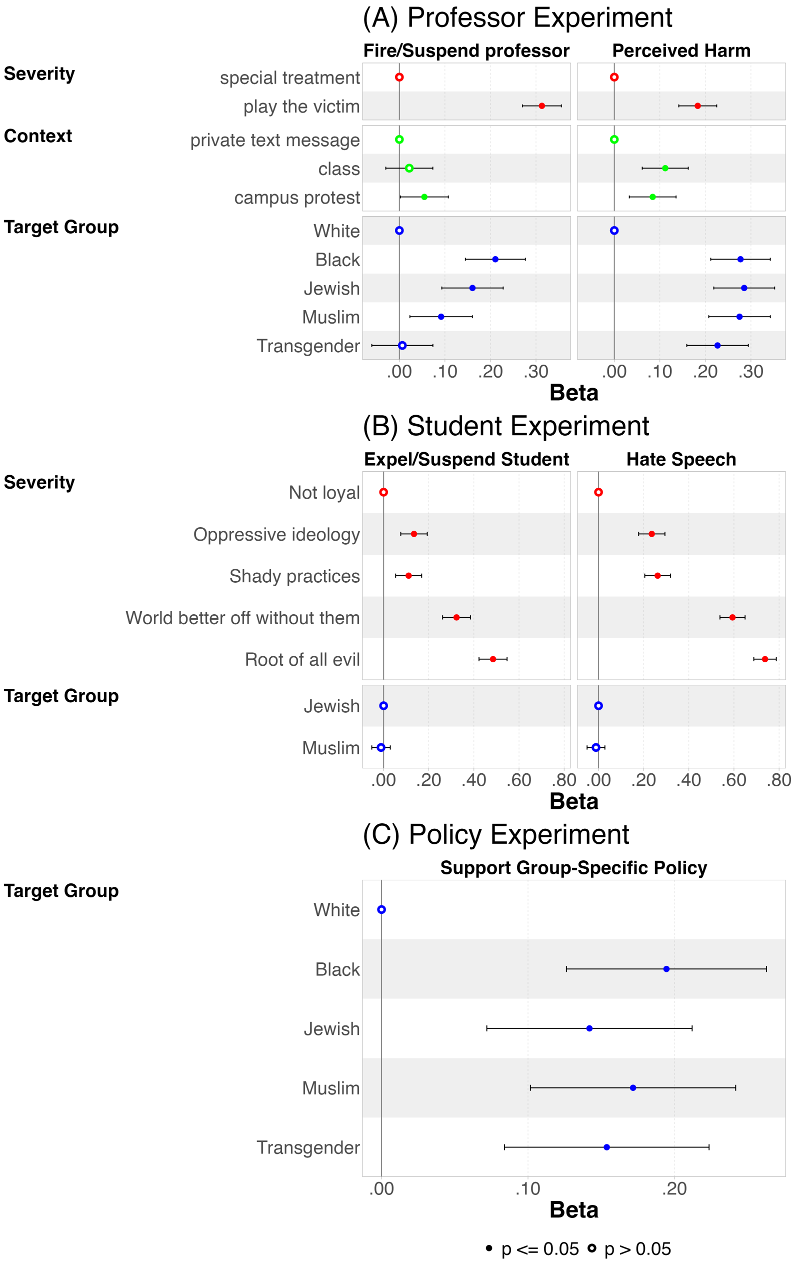

Figure S23: **Main effects by target, severity, and context (Adult Replication of of Fig. 1).** Coefficients from the pre-registered linear probability models for the three experiments: (A) *professor* (left: support for firing; right: perceived physical/psychological harm), (B) *student* (left: support for suspension/expulsion; right: hate-speech classification), and (C) *policy* (support for a rule banning offensive public statements targeting a randomized group). Dots show point estimates with 95% CIs. Reference categories: *white* targets; *"play the victim to get special treatment"* (professor severity) and *"not loyal"* (student severity); *private text* (professor context). Binary outcomes are coded as described in the Methods (e.g., firing=1 vs otherwise; suspend/expel=1 vs otherwise; hate-speech=yes=1; policy support=somewhat/strongly support=1).

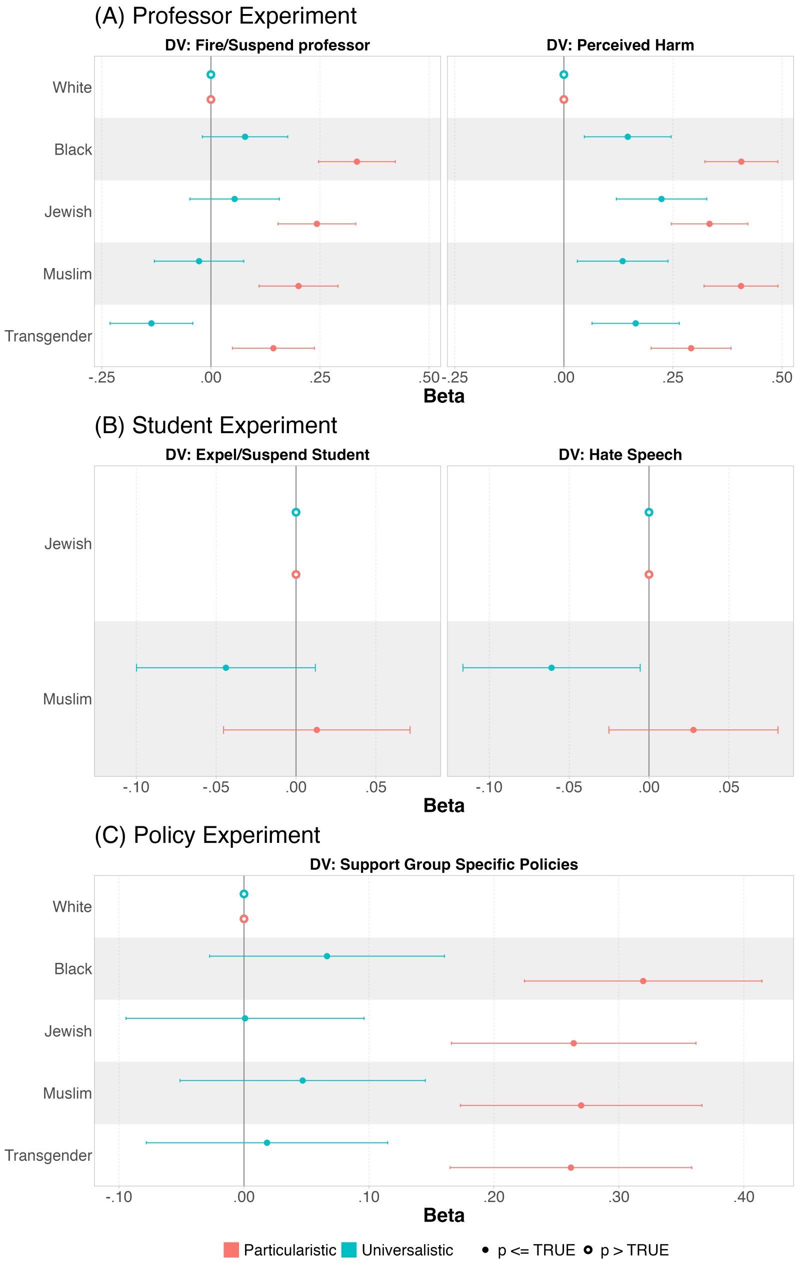

Figure S24: **Support for speech restrictions by stated principle (Adult Replication of Fig. 2).** Replicates Fig. 1's target-identity contrasts after splitting the sample by stated principle: *particularist* (rules may consider target identity) vs *universalist* (rules should not consider identity). Panels report effects on (A) firing and perceived harm in the professor experiment, (B) suspension/expulsion and hate-speech in the student experiment, and (C) support for group-specific policies. Dots show linear probability model coefficients with 95% CIs; reference categories follow the main-text specification (white targets; least severe statement; private text context)

.

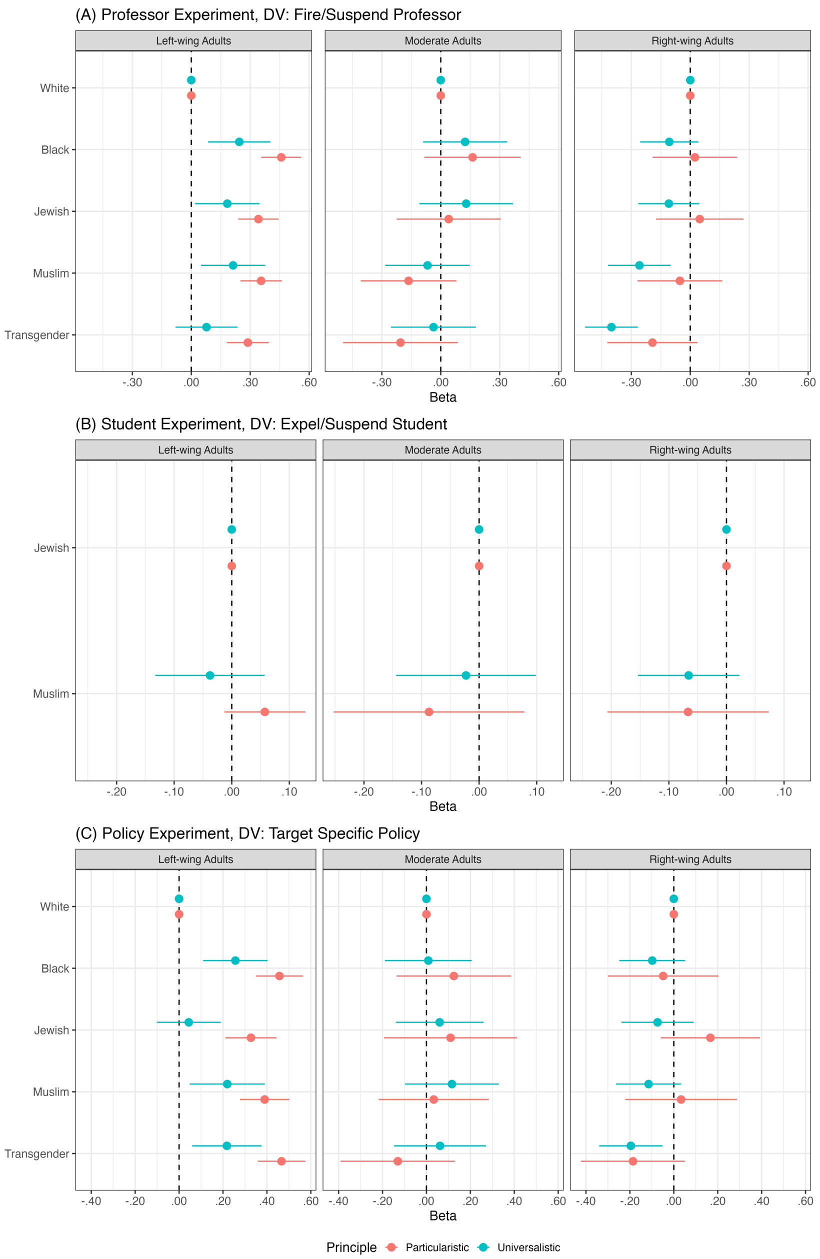

Figure S25: **Principles × political ideology (Adult replication of Fig. 3).** Estimated effects of target identity on (A) firing a professor, (B) suspending/expelling a student, and (C) supporting group-specific policy, shown separately for universalists (blue) and particularists (red) within ideological groups (left, center, right). Models match the pre-registered specifications; dots are LPM coefficients with 95% CIs . Reference categories: white targets; least severe statement; private text context.

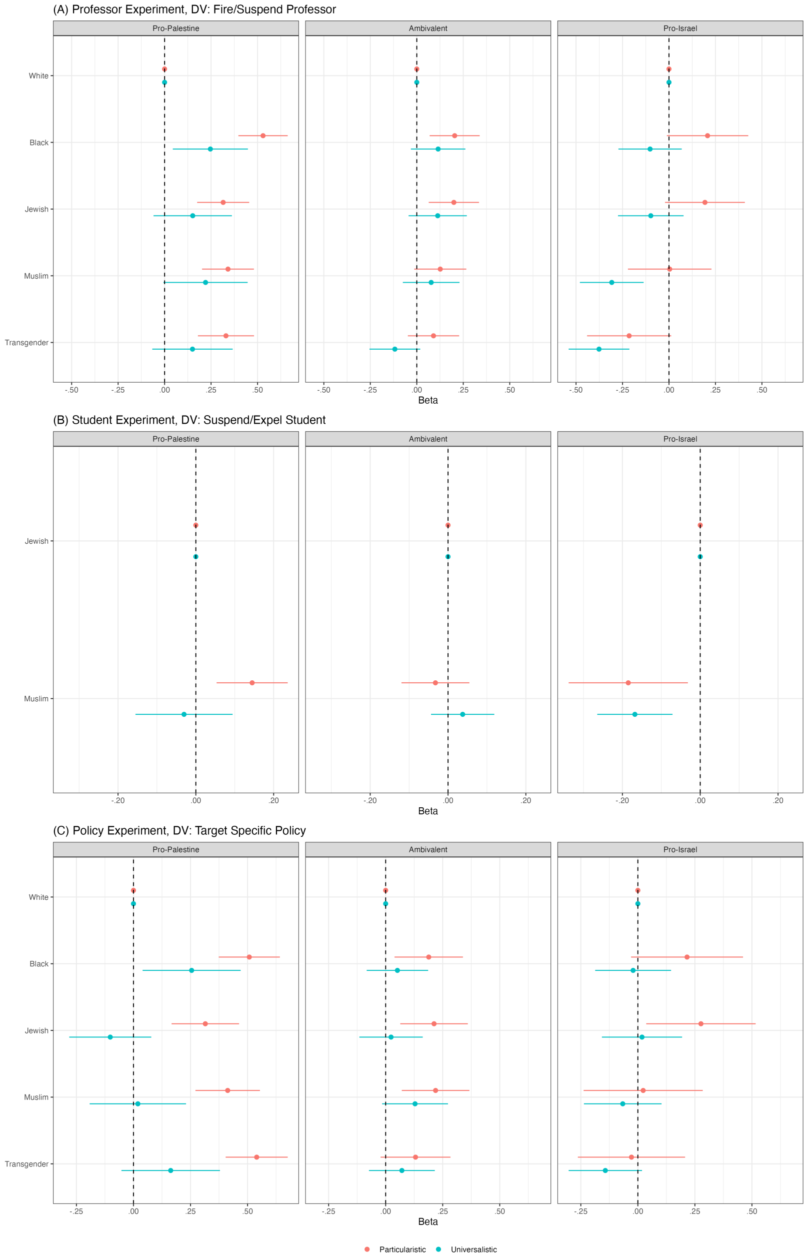

Figure S26: **Principles × Israel/Palestine sympathies (Adult replication of Fig. 4).** Estimated effects of target identity on (A) firing a professor, (B) suspending/expelling a student, and (C) supporting group-specific policy, shown separately for universalists (blue) and particularists (red) within sympathy strata defined from a continuous index: bottom quartile (more pro-Palestine), middle 50% (ambivalent), and top quartile (more pro-Israel). Models mirror the main-text specifications; dots are LPM coefficients with 95% CIs. Reference categories: white targets; least severe statement; private text context.

Supplementary Tables

Demographics

Table S1: **Demographic Distribution of Respondents to the Main Survey**

**Demographic**	**Overall (N=2966)**
**Gender**	
Female	1901 (64.1%)
Male	883 (29.8%)
Other	182 (6.1%)
**Social Class**	
Lower Class	862 (29.1%)
Middle Class	1227 (41.4%)
Upper/Upper Middle Class	877 (29.6%)
**Religion**	
protestant	247 (8.3%)
catholic	396 (13.4%)
mormon	90 (3.0%)
orthodox	37 (1.2%)
jewish	132 (4.5%)
muslim	77 (2.6%)
atheist	409 (13.8%)
agnostic	504 (17.0%)
nothing	416 (14.0%)
christian	449 (15.1%)
buddhist	45 (1.5%)
hindu	87 (2.9%)
other	77 (2.6%)
**Race**	
White	1503 (50.7%)
Black	197 (6.6%)
Asian	523 (17.6%)
Hispanic	398 (13.4%)
Other	345 (11.6%)
**Sexual Orientation**	
LGBTQ+	988 (33.3%)
Straight	1978 (66.7%)

Student Views on Speech Restrictions by Target and Severity

These tables present results from three experiments assessing support for punishing speech on university campuses. The experiments involved randomized variations in the target group and speech severity, which are estimated in separate regressions. Regression models are described in the Materials and Methods section. Labels are added for statistical significance.

Table S2: Professor Experiment

	DV: Fire/Suspend Professor		DV: Psychological Harm	
	(1)	(2)	(1)	II
Intercept	0.16***	0.16***	0.37***	0.49***
	(0.02)	(0.04)	(0.02)	(0.04)
Target Group: Black	0.33***	0.32***	0.37***	0.37***
	(0.03)	(0.03)	(0.03)	(0.03)
Target Group: Jewish	0.23***	0.22***	0.36***	0.35***
	(0.03)	(0.03)	(0.03)	(0.03)
Target Group: Muslim	0.22***	0.22***	0.33***	0.33***
	(0.03)	(0.03)	(0.03)	(0.03)
Target Group: Transgender	0.13***	0.12***	0.34***	0.33***
	(0.03)	(0.03)	(0.03)	(0.03)
Severity: Better Place Without Them	0.24***	0.25***	0.07***	0.07***
	(0.02)	(0.02)	(0.02)	(0.02)
Context: In Class	0.01	0.02	0.05**	0.05**
	(0.02)	(0.02)	(0.02)	(0.02)
Num.Obs.	2966	2966	2966	2966
R2	0.108	0.154	0.103	0.133
Controls	No	Yes	No	Yes

+ p <0.1, * p <0.05, ** p <0.01, *** p <0.001

Table S3: Student Experiment

	DV: Expel/Suspend Student		DV: Hate Speech	
	(1)	(2)	(1)	II
Intercept	0.17***	0.18***	0.13***	0.18***
	(0.02)	(0.04)	(0.02)	(0.03)
Target Group: Muslim	0.00	0.00	-0.03*	-0.03*
	(0.02)	(0.02)	(0.01)	(0.01)
Severity: Shady Practices	0.12***	0.12***	0.32***	0.32***
	(0.02)	(0.02)	(0.02)	(0.02)
Statement: Oppressive Ideology	0.05*	0.06*	0.23***	0.23***
	(0.02)	(0.02)	(0.02)	(0.02)
Statement: World Better Off Without Them	0.33***	0.32***	0.69***	0.69***
	(0.03)	(0.03)	(0.02)	(0.02)
Statement: Root of All Evil	0.45***	0.45***	0.79***	0.79***
	(0.03)	(0.02)	(0.02)	(0.02)
Num.Obs.	2966	2966	2961	2961
R2	0.127	0.155	0.349	0.365
Controls	No	Yes	No	Yes

+ p <0.1, * p <0.05, ** p <0.01, *** p <0.001

Table S4: Policy Experiment

	Support Policy	
	(1)	II
Intercept	0.29***	0.38***
	(0.02)	(0.04)
Target Group: Black	0.37***	0.38***
	(0.03)	(0.03)
Target Group: Jewish	0.33***	0.34***
	(0.03)	(0.03)
Target Group: Muslim	0.36***	0.37***
	(0.03)	(0.03)
Target Group: Transgender	0.30***	0.30***
	(0.03)	(0.03)
Num.Obs.	2966	2966
R2	0.079	0.121
Controls	No	Yes

+ p <0.1, * p <0.05, ** p <0.01, *** p <0.001

Support for Speech Restrictions by Principles

These tables present results focusing on the identity of the target of speech, but subsets the sample by respondents who adhere to particularistic or universalistic principles. It shows estimated effects on support for firing a professor, expelling a student, or endorsing group-specific policies. We include all randomized variables in the models. Regression models are described in the Materials and Methods section. Labels are added for statistical significance

Table S5: Professor Experiment. DV=Fire/Suspend Professor

	Particularistic		Universalistic	
	(1)	(2)	(1)	II
Intercept	0.15***	0.13*	0.18***	0.21**
	(0.03)	(0.05)	(0.04)	(0.07)
Target Group: Black	0.39***	0.39***	0.22***	0.21***
	(0.03)	(0.03)	(0.04)	(0.04)
Target Group: Jewish	0.30***	0.30***	0.11*	0.10*
	(0.03)	(0.03)	(0.04)	(0.04)
Target Group: Muslim	0.33***	0.33***	0.01	0.01
	(0.03)	(0.03)	(0.04)	(0.04)
Target Group: Transgender	0.20***	0.20***	-0.00	-0.02
	(0.03)	(0.03)	(0.05)	(0.04)
Severity: Better Place Without Them	0.24***	0.25***	0.26***	0.25***
	(0.02)	(0.02)	(0.03)	(0.03)
Context: In Class	0.01	0.02	0.02	0.02
	(0.03)	(0.03)	(0.03)	(0.03)
Num.Obs.	1890	1890	1076	1076
R2	0.131	0.162	0.102	0.157
Controls	No	Yes	No	Yes

+ p <0.1, * p <0.05, ** p <0.01, *** p <0.001

Table S6: Student Experiment by Principle. DV=Expel/Suspend Student

	Particularistic		Universalistic	
	(1)	(2)	(1)	II
Intercept	0.17***	0.18***	0.17***	0.22**
	(0.02)	(0.05)	(0.03)	(0.07)
Target Group: Muslim	0.01	0.02	-0.02	-0.02
	(0.02)	(0.02)	(0.03)	(0.03)
Severity: Shady Practices	0.15***	0.16***	0.05	0.06+
	(0.03)	(0.03)	(0.04)	(0.04)
Severity: Oppressive Ideology	0.08**	0.08**	0.01	0.02
	(0.03)	(0.03)	(0.03)	(0.03)
Severity: World Better Off Without Them	0.37***	0.37***	0.25***	0.25***
	(0.03)	(0.03)	(0.04)	(0.04)
Severity: Root of All Evil	0.47***	0.48***	0.39***	0.39***
	(0.03)	(0.03)	(0.04)	(0.04)
Num.Obs.	1890	1890	1076	1076
R2	0.134	0.154	0.114	0.153
Controls	No	Yes	No	Yes

+ p <0.1, * p <0.05, ** p <0.01, *** p <0.001

Table S7: Policy Experiment

	Particularistic		Universalistic	
	(1)	(2)	(1)	II
Intercept	0.25***	0.34***	0.37***	0.41***
	(0.02)	(0.05)	(0.03)	(0.07)
Target Group: Black	0.51***	0.51***	0.11*	0.12*
	(0.03)	(0.03)	(0.05)	(0.05)
Target Group: Jewish	0.45***	0.45***	0.13**	0.14**
	(0.03)	(0.03)	(0.05)	(0.05)
Target Group: Muslim	0.51***	0.52***	0.11*	0.11*
	(0.03)	(0.03)	(0.05)	(0.05)
Target Group: Transgender	0.50***	0.50***	-0.03	-0.02
	(0.03)	(0.03)	(0.05)	(0.05)
Num.Obs.	1890	1890	1076	1076
R2	0.170	0.196	0.017	0.052
Controls	No	Yes	No	Yes

+ p <0.1, * p <0.05, ** p <0.01, *** p <0.001

Support for Speech Restrictions controlling for Principles

These tables present results from the three experiments assessing support for punishing speech on university campuses and include additional controls for a person's free speech principles. Labels are added for statistical significance

Table S8: Professor Experiment

	DV: Fire/Suspend Professor		DV: Psychological Harm	
	(1)	(2)	(1)	II
Intercept	0.21***	0.18***	0.42***	0.52***
	(0.02)	(0.04)	(0.03)	(0.04)
Target Group: Black	0.33***	0.32***	0.37***	0.37***
	(0.03)	(0.03)	(0.03)	(0.03)
Target Group: Jewish	0.23***	0.22***	0.36***	0.35***
	(0.03)	(0.03)	(0.03)	(0.03)
Target Group: Muslim	0.22***	0.21***	0.33***	0.33***
	(0.03)	(0.03)	(0.03)	(0.03)
Target Group: Transgender	0.13***	0.12***	0.33***	0.33***
	(0.03)	(0.03)	(0.03)	(0.03)
Severity: Better Place Without Them	0.24***	0.25***	0.07***	0.07***
	(0.02)	(0.02)	(0.02)	(0.02)
Principles: Universalistic	-0.12***	-0.06**	-0.14***	-0.10***
	(0.02)	(0.02)	(0.02)	(0.02)
Num.Obs.	2966	2966	2966	2966
R2	0.121	0.156	0.125	0.143
Controls	No	Yes	No	Yes

+ p <0.1, * p <0.05, ** p <0.01, *** p <0.001

Table S9: Student Experiment

	DV: Expel/Suspend Student		DV: Hate Speech	
	(1)	(2)	(1)	II
Intercept	0.20***	0.20***	0.17***	0.21***
	(0.02)	(0.04)	(0.02)	(0.04)
Target Group: Muslim	0.00	0.00	-0.03*	-0.03*
	(0.02)	(0.02)	(0.01)	(0.01)
Severity: Shady Practices	0.12***	0.12***	0.32***	0.32***
	(0.02)	(0.02)	(0.02)	(0.02)
Severity: Oppressive Ideology	0.05*	0.06*	0.23***	0.23***
	(0.02)	(0.02)	(0.02)	(0.02)
Severity: World Better Off Without Them	0.32***	0.32***	0.69***	0.68***
	(0.03)	(0.03)	(0.02)	(0.02)
Severity: Root of All Evil	0.44***	0.44***	0.79***	0.78***
	(0.03)	(0.02)	(0.02)	(0.02)
Principles: Universalistic	-0.09***	-0.04*	-0.11***	-0.07***
	(0.02)	(0.02)	(0.02)	(0.02)
Num.Obs.	2966	2966	2961	2961
R2	0.135	0.157	0.359	0.369
Controls	No	Yes	No	Yes

+ p <0.1, * p <0.05, ** p <0.01, *** p <0.001

Table S10: Policy Experiment

	Support Policy	
	(1)	II
Intercept	0.37***	0.43***
	(0.02)	(0.04)
Target Group: Black	0.37***	0.37***
	(0.03)	(0.03)
Target Group: Jewish	0.33***	0.34***
	(0.03)	(0.03)
Target Group: Muslim	0.36***	0.37***
	(0.03)	(0.03)
Target Group: Transgender	0.30***	0.31***
	(0.03)	(0.03)
Principles: Universalistic	-0.20***	-0.16***
	(0.02)	(0.02)
Num.Obs.	2966	2966
R2	0.117	0.142
Controls	No	Yes

+ p <0.1, * p <0.05, ** p <0.01, *** p <0.001

Support for Speech Restrictions by Principles by Ideology (Figure 3)

Table S11: **Models examining support for firing or suspending professors.** Professor Experiment by Principle among Left-wing Respondents. DV: Fire/Suspend Professor

	Particularistic		Universalistic	
	(1)	(2)	(1)	II
Intercept	0.10**	0.07	0.03	-0.04
	(0.03)	(0.07)	(0.05)	(0.10)
Target Group: Black	0.50***	0.50***	0.38***	0.36***
	(0.04)	(0.04)	(0.07)	(0.06)
Target Group: Jewish	0.37***	0.37***	0.26***	0.22***
	(0.04)	(0.04)	(0.06)	(0.06)
Target Group: Muslim	0.42***	0.42***	0.29***	0.24***
	(0.04)	(0.04)	(0.07)	(0.07)
Target Group: Transgender	0.28***	0.28***	0.27***	0.24***
	(0.04)	(0.04)	(0.07)	(0.07)
Severity: Better Place Without Them	0.26***	0.26***	0.26***	0.27***
	(0.03)	(0.03)	(0.04)	(0.04)
Context: In Class	0.01	0.02	0.05	0.05
	(0.03)	(0.03)	(0.05)	(0.05)
Num.Obs.	1259	1259	445	445
R2	0.184	0.205	0.133	0.213
Controls	No	Yes	No	Yes

+ p <0.1, * p <0.05, ** p <0.01, *** p <0.001
Standard errors in parentheses.

Table S12: **Models examining support for firing or suspending professors.** Professor Experiment by Principle among Moderate Respondents. DV: Fire/Suspend Professor

	Particularistic		Universalistic	
	(1)	(2)	(1)	II
Intercept	0.17*	0.10	0.28***	0.36*
	(0.07)	(0.13)	(0.08)	(0.14)
Target Group: Black	0.23**	0.22**	0.28***	0.28***
	(0.08)	(0.08)	(0.08)	(0.08)
Target Group: Jewish	0.23**	0.21**	0.05	0.05
	(0.08)	(0.08)	(0.09)	(0.09)
Target Group: Muslim	0.25***	0.25**	-0.02	-0.01
	(0.07)	(0.07)	(0.09)	(0.09)
Target Group: Transgender	0.16*	0.15*	-0.17*	-0.19*
	(0.07)	(0.08)	(0.08)	(0.08)
Severity: Better Place Without Them	0.21***	0.21***	0.23***	0.23***
	(0.05)	(0.05)	(0.06)	(0.06)
Context: In Class	0.10+	0.11+	-0.04	-0.09
	(0.06)	(0.06)	(0.07)	(0.07)
Num.Obs.	422	422	288	288
R2	0.087	0.106	0.157	0.215
Controls	No	Yes	No	Yes

+ p <0.1, * p <0.05, ** p <0.01, *** p <0.001
Standard errors in parentheses.

Table S13: **Models examining support for firing or suspending professors.** Professor Experiment by Principle among Right-wing Respondents. DV: Fire/Suspend Professor

	Particularistic		Universalistic	
	(1)	(2)	(1)	II
Intercept	0.27**	0.12	0.28***	0.48***
	(0.09)	(0.18)	(0.07)	(0.14)
Target Group: Black	0.05	0.06	-0.02	-0.02
	(0.10)	(0.10)	(0.08)	(0.08)
Target Group: Jewish	0.03	0.05	-0.01	-0.03
	(0.11)	(0.11)	(0.08)	(0.08)
Target Group: Muslim	0.01	0.02	-0.26***	-0.26***
	(0.10)	(0.10)	(0.07)	(0.07)
Target Group: Transgender	-0.17+	-0.18+	-0.21**	-0.21**
	(0.10)	(0.10)	(0.08)	(0.08)
Severity: Better Place Without Them	0.24***	0.25***	0.25***	0.24***
	(0.06)	(0.06)	(0.05)	(0.05)
Context: In Class	-0.07	-0.06	0.02	0.03
	(0.08)	(0.08)	(0.06)	(0.06)
Num.Obs.	209	209	343	343
R2	0.084	0.109	0.138	0.156
Controls	No	Yes	No	Yes

+ p <0.1, * p <0.05, ** p <0.01, *** p <0.001
Standard errors in parentheses.

Table S14: **Models examining support for expelling or suspending students.** Student Experiment by Principle among Left-wing Respondents. DV: Expel/Suspend Student

	Particularistic		Universalistic	
	(1)	(2)	(1)	II
Intercept	0.17***	0.12+	0.23***	0.27**
	(0.03)	(0.06)	(0.05)	(0.09)
Target Group: Muslim	0.06*	0.06*	-0.02	-0.02
	(0.03)	(0.03)	(0.04)	(0.04)
Statement: Oppressive Ideology	0.06+	0.08*	-0.09	-0.07
	(0.04)	(0.04)	(0.06)	(0.06)
Statement: Shady Practices	0.16***	0.17***	0.04	0.04
	(0.04)	(0.04)	(0.06)	(0.06)
Statement: World Better Off Without Them	0.39***	0.39***	0.25***	0.24***
	(0.04)	(0.04)	(0.07)	(0.07)
Statement: Root of All Evil	0.50***	0.51***	0.44***	0.43***
	(0.04)	(0.04)	(0.07)	(0.07)
Num.Obs.	1259	1259	445	445
R2	0.156	0.173	0.157	0.190
Controls	No	Yes	No	Yes

+ p <0.1, * p <0.05, ** p <0.01, *** p <0.001
Standard errors in parentheses.

Table S15: **Models examining support for expelling or suspending students.** Student Experiment by Principle among Moderate Respondents. DV: Expel/Suspend Student

	Particularistic		Universalistic	
	(1)	(2)	(1)	II
Intercept	0.17***	0.22*	0.20**	0.09
	(0.04)	(0.10)	(0.06)	(0.12)
Target Group: Muslim	-0.06	-0.06	-0.00	0.01
	(0.04)	(0.05)	(0.05)	(0.06)
Statement: Oppressive Ideology	0.12+	0.11+	0.07	0.06
	(0.06)	(0.06)	(0.07)	(0.07)
Statement: Shady Practices	0.14*	0.15*	0.05	0.05
	(0.06)	(0.06)	(0.08)	(0.08)
Statement: World Better Off Without Them	0.36***	0.35***	0.25**	0.25**
	(0.07)	(0.07)	(0.09)	(0.09)
Statement: Root of All Evil	0.39***	0.40***	0.32***	0.31***
	(0.07)	(0.07)	(0.09)	(0.09)
Num.Obs.	422	422	288	288
R2	0.097	0.122	0.067	0.106
Controls	No	Yes	No	Yes

+ p <0.1, * p <0.05, ** p <0.01, *** p <0.001
Standard errors in parentheses.

Table S16: **Models examining support for expelling or suspending students.** Student Experiment by Principle among Right-wing Respondents. DV: Expel/Suspend Student

	Particularistic		Universalistic	
	(1)	(2)	(1)	II
Intercept	0.19**	0.06	0.06	0.09
	(0.06)	(0.13)	(0.04)	(0.12)
Target Group: Muslim	-0.12+	-0.15*	-0.02	-0.03
	(0.06)	(0.06)	(0.04)	(0.04)
Statement: Oppressive Ideology	0.08	0.05	0.06	0.06
	(0.08)	(0.08)	(0.04)	(0.05)
Statement: Shady Practices	0.11	0.09	0.08+	0.10*
	(0.08)	(0.09)	(0.05)	(0.05)
Statement: World Better Off Without Them	0.25**	0.22*	0.23***	0.22***
	(0.09)	(0.09)	(0.06)	(0.06)
Statement: Root of All Evil	0.46***	0.45***	0.37***	0.38***
	(0.09)	(0.09)	(0.07)	(0.07)
Num.Obs.	209	209	343	343
R2	0.143	0.168	0.117	0.132
Controls	No	Yes	No	Yes

+ p <0.1, * p <0.05, ** p <0.01, *** p <0.001
Standard errors in parentheses.

Table S17: **Models examining support for targeted campus policies.** Target Specific Policies by Principle among Left--Wing Respondents

	Particularistic		Universalistic	
	(1)	(2)	(1)	II
Intercept	0.18***	0.22***	0.30***	0.24*
	(0.02)	(0.06)	(0.05)	(0.10)
Target Group: Black	0.62***	0.62***	0.26***	0.26***
	(0.03)	(0.03)	(0.07)	(0.07)
Target Group: Jewish	0.57***	0.57***	0.28***	0.28***
	(0.04)	(0.04)	(0.07)	(0.07)
Target Group: Muslim	0.62***	0.64***	0.28***	0.29***
	(0.04)	(0.04)	(0.07)	(0.07)
Target Group: Transgender	0.64***	0.64***	0.16*	0.17*
	(0.03)	(0.04)	(0.07)	(0.07)
Num.Obs.	1259	1259	445	445
R2	0.266	0.284	0.046	0.101
Controls	No	Yes	No	Yes

+ p <0.1, * p <0.05, ** p <0.01, *** p <0.001
Standard errors in parentheses.

Table S18: **Models examining support for targeted campus policies.** Target Specific Policies by Principle among Moderate Respondents

	Particularistic		Universalistic	
	(1)	(2)	(1)	II
Intercept	0.30***	0.44***	0.47***	0.46**
	(0.05)	(0.11)	(0.07)	(0.14)
Target Group: Black	0.46***	0.45***	-0.02	-0.03
	(0.07)	(0.07)	(0.10)	(0.10)
Target Group: Jewish	0.28***	0.28***	-0.00	-0.00
	(0.07)	(0.07)	(0.09)	(0.10)
Target Group: Muslim	0.33***	0.33***	0.02	0.01
	(0.07)	(0.07)	(0.10)	(0.10)
Target Group: Transgender	0.33***	0.33***	-0.20*	-0.20*
	(0.07)	(0.07)	(0.09)	(0.09)
Num.Obs.	422	422	288	288
R2	0.096	0.118	0.029	0.070
Controls	No	Yes	No	Yes

+ p <0.1, * p <0.05, ** p <0.01, *** p <0.001
Standard errors in parentheses.

Table S19: **Models examining support for targeted campus policies.** Target Specific Policies by Principle among Right-Wing Respondents

	Particularistic		Universalistic	
	(1)	(2)	(1)	II
Intercept	0.50***	0.44*	0.39***	0.41**
	(0.07)	(0.17)	(0.06)	(0.13)
Target Group: Black	0.05	0.06	0.03	0.05
	(0.10)	(0.10)	(0.08)	(0.08)
Target Group: Jewish	0.14	0.14	0.05	0.05
	(0.10)	(0.10)	(0.08)	(0.08)
Target Group: Muslim	0.22*	0.23*	-0.06	-0.04
	(0.10)	(0.10)	(0.08)	(0.08)
Target Group: Transgender	-0.05	-0.03	-0.12	-0.11
	(0.12)	(0.12)	(0.08)	(0.08)
Num.Obs.	209	209	343	343
R2	0.036	0.113	0.017	0.031
Controls	No	Yes	No	Yes

+ p <0.1, * p <0.05, ** p <0.01, *** p <0.001
Standard errors in parentheses.

Support for Speech Restrictions by Principles by Israel-Palestine Sympathy (Figure 4)

Table S20: **Models examining support for firing or suspending professors.** Professor Experiment by Principle among Those More Sympathetic to Palestinians. DV: Fire/Suspend Professor

	Particularistic		Universalistic	
	(1)	(2)	(1)	II
Intercept	0.05	0.04	0.03	0.15
	(0.05)	(0.06)	(0.09)	(0.12)
Target Group: Black	0.67***	0.67***	0.51***	0.53***
	(0.05)	(0.05)	(0.10)	(0.10)
Target Group: Jewish	0.42***	0.42***	0.21+	0.19+
	(0.05)	(0.05)	(0.11)	(0.11)
Target Group: Muslim	0.60***	0.60***	0.33**	0.31*
	(0.05)	(0.05)	(0.12)	(0.12)
Target Group: Transgender	0.47***	0.46***	0.26*	0.27*
	(0.06)	(0.06)	(0.11)	(0.11)
Severity: Better Place Without Them	0.23***	0.22***	0.37***	0.39***
	(0.03)	(0.04)	(0.07)	(0.07)
Context: In Class	0.02	0.02	0.01	0.01
	(0.04)	(0.04)	(0.08)	(0.08)
Num.Obs.	592	592	161	161
R2	0.271	0.285	0.267	0.313
Controls	No	Yes	No	Yes

+ p <0.1, * p <0.05, ** p <0.01, *** p <0.001
Standard errors in parentheses.

Table S21: **Models examining support for firing or suspending professors.** Professor Experiment by Principle among Ambivalent Respondents. DV: Fire/Suspend Professor

	Particularistic		Universalistic	
	(1)	(2)	(1)	II
Intercept	0.15***	0.17**	0.17***	0.18*
	(0.04)	(0.06)	(0.05)	(0.08)
Target Group: Black	0.33***	0.32***	0.23***	0.24***
	(0.04)	(0.04)	(0.06)	(0.05)
Target Group: Jewish	0.27***	0.26***	0.13*	0.12*
	(0.04)	(0.04)	(0.06)	(0.06)
Target Group: Muslim	0.28***	0.27***	0.06	0.05
	(0.04)	(0.04)	(0.06)	(0.06)
Target Group: Transgender	0.15**	0.14**	0.03	0.03
	(0.05)	(0.05)	(0.06)	(0.06)
Severity: Better Place Without Them	0.27***	0.27***	0.26***	0.25***
	(0.03)	(0.03)	(0.04)	(0.04)
Context: In Class	0.02	0.03	-0.02	-0.03
	(0.04)	(0.03)	(0.05)	(0.05)
Num.Obs.	1039	1039	627	627
R2	0.131	0.155	0.102	0.158
Controls	No	Yes	No	Yes

+ p <0.1, * p <0.05, ** p <0.01, *** p <0.001
Standard errors in parentheses.

Table S22: **Models examining support for firing or suspending professors.** Professor Experiment by Principle among Those More Sympathetic to Israelis. DV: Fire/Suspend Professor

	Particularistic		Universalistic	
	(1)	(2)	(1)	II
Intercept	0.35***	0.30*	0.26**	0.29*
	(0.09)	(0.14)	(0.08)	(0.14)
Target Group: Black	0.06	0.06	0.01	0.02
	(0.10)	(0.10)	(0.09)	(0.09)
Target Group: Jewish	0.14	0.13	0.03	0.01
	(0.10)	(0.10)	(0.09)	(0.09)
Target Group: Muslim	-0.08	-0.08	-0.22**	-0.21*
	(0.09)	(0.10)	(0.08)	(0.08)
Target Group: Transgender	-0.15	-0.12	-0.20*	-0.23**
	(0.10)	(0.10)	(0.09)	(0.09)
Severity: Better Place Without Them	0.20**	0.20**	0.22***	0.19***
	(0.06)	(0.06)	(0.05)	(0.05)
Context: In Class	0.02	0.02	0.10	0.09
	(0.07)	(0.08)	(0.07)	(0.07)
Num.Obs.	259	259	288	288
R2	0.068	0.118	0.112	0.166
Controls	No	Yes	No	Yes

+ p <0.1, * p <0.05, ** p <0.01, *** p <0.001
Standard errors in parentheses.

Table S23: **Models examining support for expelling or suspending students.** Student Experiment by Principle among Those More Sympathetic to Palestinians. DV: Expel/Suspend Student

	Particularistic		Universalistic	
	(1)	(2)	(1)	II
Intercept	0.19***	0.24***	0.22**	0.25*
	(0.04)	(0.07)	(0.08)	(0.12)
Target Group: Muslim	0.10**	0.10**	0.09	0.08
	(0.04)	(0.04)	(0.07)	(0.07)
Statement: Oppressive Ideology	0.02	0.03	-0.16+	-0.12
	(0.06)	(0.06)	(0.09)	(0.10)
Statement: Shady Practices	0.21***	0.21***	0.09	0.15
	(0.06)	(0.06)	(0.12)	(0.13)
Statement: World Better Off Without Them	0.38***	0.39***	0.37**	0.36**
	(0.06)	(0.06)	(0.11)	(0.12)
Statement: Root of All Evil	0.55***	0.55***	0.40***	0.43***
	(0.05)	(0.05)	(0.11)	(0.11)
Num.Obs.	592	592	161	161
R2	0.194	0.210	0.200	0.248
Controls	No	Yes	No	Yes

+ p <0.1, * p <0.05, ** p <0.01, *** p <0.001
Standard errors in parentheses.

Table S24: **Models examining support for expelling or suspending students.** Student Experiment by Principle among Ambivalent Respondents. DV: Expel/Suspend Student

	Particularistic		Universalistic	
	(1)	(2)	(1)	II
Intercept	0.15***	0.13**	0.19***	0.31***
	(0.03)	(0.05)	(0.04)	(0.07)
Target Group: Muslim	-0.02	-0.02	-0.04	-0.05
	(0.03)	(0.03)	(0.03)	(0.03)
Statement: Oppressive Ideology	0.12**	0.11**	0.04	0.05
	(0.04)	(0.04)	(0.05)	(0.05)
Statement: Shady Practices	0.14***	0.13**	0.03	0.05
	(0.04)	(0.04)	(0.05)	(0.05)
Statement: World Better Off Without Them	0.36***	0.36***	0.18**	0.18**
	(0.04)	(0.04)	(0.06)	(0.06)
Statement: Root of All Evil	0.44***	0.43***	0.38***	0.40***
	(0.04)	(0.04)	(0.06)	(0.06)
Num.Obs.	1039	1039	627	627
R2	0.117	0.127	0.099	0.131
Controls	No	Yes	No	Yes

+ p <0.1, * p <0.05, ** p <0.01, *** p <0.001
Standard errors in parentheses.

Table S25: **Models examining support for expelling or suspending students.** Student Experiment by Principle among Those More Sympathetic to Israelis. DV: Expel/Suspend Student

	Particularistic		Universalistic	
	(1)	(2)	(1)	II
Intercept	0.21**	0.14	0.10*	-0.00
	(0.06)	(0.12)	(0.05)	(0.11)
Target Group: Muslim	-0.06	-0.08	-0.03	-0.03
	(0.06)	(0.06)	(0.05)	(0.05)
Statement: Oppressive Ideology	0.10	0.10	0.03	0.04
	(0.08)	(0.08)	(0.05)	(0.05)
Statement: Shady Practices	0.11	0.10	0.05	0.11+
	(0.09)	(0.09)	(0.06)	(0.07)
Statement: World Better Off Without Them	0.35***	0.34***	0.30***	0.31***
	(0.08)	(0.09)	(0.07)	(0.07)
Statement: Root of All Evil	0.46***	0.45***	0.37***	0.39***
	(0.09)	(0.09)	(0.08)	(0.08)
Num.Obs.	259	259	288	288
R2	0.128	0.158	0.137	0.180
Controls	No	Yes	No	Yes

+ p <0.1, * p <0.05, ** p <0.01, *** p <0.001
Standard errors in parentheses.

Table S26: **Models examining support for targeted campus policies.** Target Specific Policies by Principle among Those More Sympathetic to Palestinians

	Particularistic		Universalistic	
	(1)	(2)	(1)	II
Intercept	0.13***	0.18***	0.27**	0.38***
	(0.03)	(0.05)	(0.08)	(0.11)
Target Group: Black	0.74***	0.74***	0.46***	0.47***
	(0.04)	(0.04)	(0.11)	(0.12)
Target Group: Jewish	0.60***	0.60***	0.32*	0.33*
	(0.05)	(0.05)	(0.12)	(0.13)
Target Group: Muslim	0.72***	0.73***	0.30*	0.33*
	(0.05)	(0.05)	(0.12)	(0.13)
Target Group: Transgender	0.71***	0.72***	0.38**	0.38**
	(0.05)	(0.05)	(0.12)	(0.12)
Num.Obs.	592	592	161	161
R2	0.356	0.379	0.096	0.155
Controls	No	Yes	No	Yes

+ p <0.1, * p <0.05, ** p <0.01, *** p <0.001
Standard errors in parentheses.

Table S27: **Models examining support for targeted campus policies.** Target Specific Policies by Principle among Ambivalent Respondents

	Particularistic		Universalistic	
	(1)	(2)	(1)	II
Intercept	0.25***	0.25***	0.35***	0.33***
	(0.03)	(0.05)	(0.05)	(0.07)
Target Group: Black	0.44***	0.44***	0.06	0.07
	(0.04)	(0.04)	(0.06)	(0.07)
Target Group: Jewish	0.40***	0.41***	0.10	0.12+
	(0.04)	(0.04)	(0.06)	(0.06)
Target Group: Muslim	0.45***	0.47***	0.17**	0.18**
	(0.04)	(0.04)	(0.06)	(0.06)
Target Group: Transgender	0.45***	0.45***	-0.03	-0.01
	(0.04)	(0.04)	(0.06)	(0.06)
Num.Obs.	1039	1039	627	627
R2	0.130	0.152	0.022	0.059
Controls	No	Yes	No	Yes

+ p <0.1, * p <0.05, ** p <0.01, *** p <0.001
Standard errors in parentheses.

Table S28: **Models examining support for targeted campus policies.** Target Specific Policies by Principle among Those More Sympathetic to Israelis

	Particularistic		Universalistic	
	(1)	(2)	(1)	II
Intercept	0.48***	0.52***	0.45***	0.45**
	(0.07)	(0.12)	(0.06)	(0.15)
Target Group: Black	0.31***	0.30***	0.04	0.06
	(0.09)	(0.09)	(0.09)	(0.09)
Target Group: Jewish	0.28**	0.28**	0.11	0.13
	(0.09)	(0.09)	(0.09)	(0.09)
Target Group: Muslim	0.22*	0.22*	-0.14	-0.13
	(0.10)	(0.10)	(0.09)	(0.09)
Target Group: Transgender	0.23*	0.22*	-0.22**	-0.21*
	(0.10)	(0.10)	(0.08)	(0.09)
Num.Obs.	259	259	288	288
R2	0.058	0.093	0.058	0.100
Controls	No	Yes	No	Yes

+ p <0.1, * p <0.05, ** p <0.01, *** p <0.001
Standard errors in parentheses.

Figure 3 Results with Harm DV

Table S29: **Models examining perceived psychological harm from professor speech.** Professor Experiment by Principle among Left-wing Respondents. DV: Psychological Harm

	Particularistic		Universalistic	
	(1)	(2)	(1)	II
Intercept	0.29***	0.37***	0.35***	0.28*
	(0.03)	(0.06)	(0.06)	(0.11)
Target Group: Black	0.58***	0.58***	0.40***	0.40***
	(0.03)	(0.04)	(0.07)	(0.07)
Target Group: Jewish	0.56***	0.56***	0.36***	0.34***
	(0.04)	(0.04)	(0.07)	(0.07)
Target Group: Muslim	0.54***	0.54***	0.31***	0.29***
	(0.04)	(0.04)	(0.07)	(0.07)
Target Group: Transgender	0.58***	0.58***	0.32***	0.31***
	(0.03)	(0.04)	(0.07)	(0.07)
Severity: Better Place Without Them	0.07***	0.07***	0.08+	0.09*
	(0.02)	(0.02)	(0.04)	(0.04)
Context: In Class	0.00	0.00	0.05	0.06
	(0.02)	(0.02)	(0.05)	(0.05)
Num.Obs.	1259	1259	445	445
R2	0.298	0.305	0.104	0.125
Controls	No	Yes	No	Yes

+ p <0.1, * p <0.05, ** p <0.01, *** p <0.001
Standard errors in parentheses.

Table S30: **Models examining perceived psychological harm from professor speech.** Professor Experiment by Principle among Moderate Respondents. DV: Psychological Harm

	Particularistic		Universalistic	
	(1)	(2)	(1)	II
Intercept	0.43***	0.58***	0.44***	0.56***
	(0.07)	(0.12)	(0.08)	(0.15)
Target Group: Black	0.38***	0.39***	0.08	0.06
	(0.07)	(0.07)	(0.09)	(0.09)
Target Group: Jewish	0.25**	0.26***	0.28**	0.29**
	(0.08)	(0.08)	(0.09)	(0.09)
Target Group: Muslim	0.33***	0.33***	0.16+	0.16
	(0.07)	(0.07)	(0.10)	(0.10)
Target Group: Transgender	0.36***	0.36***	-0.02	-0.04
	(0.07)	(0.07)	(0.09)	(0.10)
Severity: Better Place Without Them	0.07	0.07+	0.04	0.02
	(0.04)	(0.04)	(0.06)	(0.06)
Context: In Class	0.04	0.03	0.09	0.07
	(0.05)	(0.05)	(0.07)	(0.07)
Num.Obs.	422	422	288	288
R2	0.102	0.118	0.060	0.099
Controls	No	Yes	No	Yes

+ p <0.1, * p <0.05, ** p <0.01, *** p <0.001
Standard errors in parentheses.

Table S31: **Models examining perceived psychological harm from professor speech.** Professor Experiment by Principle among Right-wing Respondents. DV: Psychological Harm

	Particularistic		Universalistic	
	(1)	(2)	(1)	II
Intercept	0.40***	0.53**	0.44***	0.65***
	(0.10)	(0.18)	(0.08)	(0.15)
Target Group: Black	0.09	0.10	-0.00	0.03
	(0.11)	(0.11)	(0.08)	(0.09)
Target Group: Jewish	0.05	0.05	0.03	0.04
	(0.11)	(0.11)	(0.08)	(0.08)
Target Group: Muslim	0.16	0.16	-0.12	-0.12
	(0.10)	(0.10)	(0.08)	(0.08)
Target Group: Transgender	0.20+	0.19+	-0.19*	-0.19*
	(0.10)	(0.11)	(0.09)	(0.09)
Severity: Better Place Without Them	0.07	0.06	0.17**	0.16**
	(0.07)	(0.07)	(0.05)	(0.05)
Context: In Class	0.16*	0.17*	0.15*	0.13*
	(0.08)	(0.09)	(0.06)	(0.06)
Num.Obs.	209	209	343	343
R2	0.048	0.075	0.071	0.107
Controls	No	Yes	No	Yes

+ p <0.1, * p <0.05, ** p <0.01, *** p <0.001
Standard errors in parentheses.

Table S32: **Models examining whether student speech is classified as hate speech.** Student Experiment by Principle among Left-wing Respondents. DV: Hate Speech

	Particularistic		Universalistic	
	(1)	(2)	(1)	II
Intercept	0.11***	0.16**	0.15***	0.21*
	(0.02)	(0.06)	(0.04)	(0.10)
Target Group: Muslim	0.00	0.00	-0.01	-0.02
	(0.02)	(0.02)	(0.04)	(0.04)
Statement: Oppressive Ideology	0.29***	0.30***	0.09	0.08
	(0.04)	(0.04)	(0.06)	(0.06)
Statement: Shady Practices	0.45***	0.45***	0.26***	0.26***
	(0.04)	(0.04)	(0.06)	(0.06)
Statement: World Better Off Without Them	0.77***	0.77***	0.57***	0.56***
	(0.03)	(0.03)	(0.06)	(0.06)
Statement: Root of All Evil	0.85***	0.85***	0.78***	0.77***
	(0.02)	(0.02)	(0.05)	(0.05)
Num.Obs.	1258	1258	444	444
R2	0.400	0.406	0.347	0.359
Controls	No	Yes	No	Yes

+ p <0.1, * p <0.05, ** p <0.01, *** p <0.001
Standard errors in parentheses.

Table S33: **Models examining whether student speech is classified as hate speech.** Student Experiment by Principle among Moderate Respondents. DV: Hate Speech

	Particularistic		Universalistic	
	(1)	(2)	(1)	II
Intercept	0.18***	0.24*	0.07+	0.19
	(0.04)	(0.10)	(0.04)	(0.12)
Target Group: Muslim	-0.06	-0.07+	0.03	0.04
	(0.04)	(0.04)	(0.05)	(0.05)
Statement: Oppressive Ideology	0.20**	0.17**	0.22**	0.24***
	(0.07)	(0.07)	(0.07)	(0.07)
Statement: Shady Practices	0.15*	0.15*	0.27***	0.29***
	(0.06)	(0.06)	(0.07)	(0.08)
Statement: World Better Off Without Them	0.67***	0.66***	0.70***	0.70***
	(0.06)	(0.06)	(0.07)	(0.07)
Statement: Root of All Evil	0.76***	0.75***	0.71***	0.74***
	(0.05)	(0.05)	(0.07)	(0.07)
Num.Obs.	422	422	286	286
R2	0.361	0.393	0.307	0.352
Controls	No	Yes	No	Yes

+ p <0.1, * p <0.05, ** p <0.01, *** p <0.001
Standard errors in parentheses.

Table S34: **Models examining whether student speech is classified as hate speech.** Student Experiment by Principle among Right-wing Respondents. DV: Hate Speech

	Particularistic		Universalistic	
	(1)	(2)	(1)	II
Intercept	0.13*	-0.08	0.16***	0.18
	(0.05)	(0.12)	(0.04)	(0.11)
Target Group: Muslim	-0.10+	-0.09	-0.17***	-0.17***
	(0.06)	(0.06)	(0.05)	(0.05)
Statement: Oppressive Ideology	0.23**	0.21*	0.18**	0.17**
	(0.08)	(0.09)	(0.06)	(0.06)
Statement: Shady Practices	0.31***	0.29**	0.21***	0.19**
	(0.09)	(0.09)	(0.06)	(0.06)
Statement: World Better Off Without Them	0.68***	0.70***	0.55***	0.55***
	(0.08)	(0.07)	(0.07)	(0.07)
Statement: Root of All Evil	0.75***	0.73***	0.68***	0.67***
	(0.07)	(0.07)	(0.06)	(0.06)
Num.Obs.	209	209	342	342
R2	0.345	0.385	0.301	0.314
Controls	No	Yes	No	Yes

+ p <0.1, * p <0.05, ** p <0.01, *** p <0.001
Standard errors in parentheses.

Figure 4 Results with Hate DV

Table S35: **Models examining perceived psychological harm from professor speech.** Professor Experiment by Principle among Those More Sympathetic to Palestinians. DV: Psychological Harm

	Particularistic		Universalistic	
	(1)	(2)	(1)	II
Intercept	0.20***	0.23***	0.20*	0.16
	(0.04)	(0.06)	(0.09)	(0.10)
Target Group: Black	0.70***	0.71***	0.56***	0.54***
	(0.05)	(0.05)	(0.10)	(0.11)
Target Group: Jewish	0.62***	0.63***	0.52***	0.53***
	(0.05)	(0.05)	(0.12)	(0.11)
Target Group: Muslim	0.70***	0.71***	0.48***	0.47***
	(0.05)	(0.05)	(0.11)	(0.12)
Target Group: Transgender	0.71***	0.71***	0.38**	0.37**
	(0.05)	(0.05)	(0.11)	(0.11)
Severity: Better Place Without Them	0.04	0.04	0.12+	0.12+
	(0.03)	(0.03)	(0.06)	(0.07)
Context: In Class	0.00	0.00	-0.00	0.03
	(0.03)	(0.03)	(0.08)	(0.08)
Num.Obs.	592	592	161	161
R2	0.417	0.432	0.224	0.286
Controls	No	Yes	No	Yes

+ p <0.1, * p <0.05, ** p <0.01, *** p <0.001
Standard errors in parentheses.

Tables for additional experiments

Table S36: **Models examining perceived psychological harm from professor speech.** Professor Experiment by Principle among Ambivalent Respondents. DV: Psychological Harm

	Particularistic		Universalistic	
	(1)	(2)	(1)	II
Intercept	0.39***	0.47***	0.42***	0.55***
	(0.04)	(0.06)	(0.06)	(0.09)
Target Group: Black	0.40***	0.40***	0.17**	0.18**
	(0.04)	(0.04)	(0.06)	(0.06)
Target Group: Jewish	0.35***	0.35***	0.20***	0.19**
	(0.04)	(0.04)	(0.06)	(0.06)
Target Group: Muslim	0.37***	0.37***	0.13*	0.11+
	(0.04)	(0.05)	(0.06)	(0.06)
Target Group: Transgender	0.41***	0.41***	0.07	0.06
	(0.04)	(0.04)	(0.06)	(0.06)
Severity: Better Place Without Them	0.07**	0.07**	0.07+	0.06
	(0.03)	(0.03)	(0.04)	(0.04)
Context: In Class	0.04	0.04	0.09+	0.09+
	(0.03)	(0.03)	(0.05)	(0.05)
Num.Obs.	1039	1039	627	627
R2	0.129	0.149	0.041	0.078
Controls	No	Yes	No	Yes

+ p <0.1, * p <0.05, ** p <0.01, *** p <0.001
Standard errors in parentheses.

Table S37: **Models examining perceived psychological harm from professor speech.** Professor Experiment by Principle among Those More Sympathetic to Israelis. DV: Psychological Harm

	Particularistic		Universalistic	
	(1)	(2)	(1)	II
Intercept	0.48***	0.57***	0.44***	0.67***
	(0.09)	(0.12)	(0.08)	(0.17)
Target Group: Black	0.32***	0.30***	-0.02	0.00
	(0.09)	(0.09)	(0.09)	(0.09)
Target Group: Jewish	0.37***	0.35***	0.15+	0.16+
	(0.09)	(0.09)	(0.09)	(0.09)
Target Group: Muslim	0.17+	0.16+	-0.05	-0.05
	(0.10)	(0.10)	(0.09)	(0.09)
Target Group: Transgender	0.24**	0.24*	-0.11	-0.12
	(0.09)	(0.10)	(0.09)	(0.10)
Severity: Better Place Without Them	0.09+	0.12*	0.15**	0.14*
	(0.05)	(0.05)	(0.06)	(0.06)
Context: In Class	0.08	0.09	0.14*	0.12+
	(0.06)	(0.06)	(0.07)	(0.07)
Num.Obs.	259	259	288	288
R2	0.109	0.170	0.074	0.124
Controls	No	Yes	No	Yes

+ p <0.1, * p <0.05, ** p <0.01, *** p <0.001
Standard errors in parentheses.

Table S38: **Models examining whether student speech is classified as hate speech.** Student Experiment by Principle among Those More Sympathetic to Palestinians. DV: Hate Speech

	Particularistic		Universalistic	
	(1)	(2)	(1)	II
Intercept	0.08**	0.04	0.14+	0.14
	(0.03)	(0.05)	(0.07)	(0.09)
Target Group: Muslim	0.04	0.04	0.08	0.10
	(0.03)	(0.03)	(0.06)	(0.06)
Statement: Oppressive Ideology	0.30***	0.30***	0.10	0.10
	(0.05)	(0.05)	(0.10)	(0.11)
Statement: Shady Practices	0.45***	0.45***	0.31**	0.28*
	(0.05)	(0.05)	(0.11)	(0.11)
Statement: World Better Off Without Them	0.79***	0.79***	0.67***	0.64***
	(0.04)	(0.04)	(0.09)	(0.10)
Statement: Root of All Evil	0.88***	0.87***	0.73***	0.71***
	(0.03)	(0.03)	(0.08)	(0.09)
Num.Obs.	592	592	161	161
R2	0.451	0.459	0.370	0.399
Controls	No	Yes	No	Yes

+ p <0.1, * p <0.05, ** p <0.01, *** p <0.001
Standard errors in parentheses.

Table S39: **Models examining whether student speech is classified as hate speech.** Student Experiment by Principle among Ambivalent Respondents. DV: Hate Speech

	Particularistic		Universalistic	
	(1)	(2)	(1)	II
Intercept	0.13***	0.18***	0.10**	0.27***
	(0.03)	(0.04)	(0.03)	(0.07)
Target Group: Muslim	-0.03	-0.03	-0.05	-0.05
	(0.02)	(0.02)	(0.03)	(0.03)
Statement: Oppressive Ideology	0.24***	0.23***	0.19***	0.20***
	(0.04)	(0.04)	(0.05)	(0.05)
Statement: Shady Practices	0.33***	0.33***	0.26***	0.26***
	(0.04)	(0.04)	(0.05)	(0.05)
Statement: World Better Off Without Them	0.72***	0.72***	0.61***	0.60***
	(0.03)	(0.03)	(0.05)	(0.05)
Statement: Root of All Evil	0.81***	0.79***	0.76***	0.76***
	(0.03)	(0.03)	(0.04)	(0.04)
Num.Obs.	1038	1038	625	625
R2	0.364	0.377	0.307	0.331
Controls	No	Yes	No	Yes

+ p <0.1, * p <0.05, ** p <0.01, *** p <0.001
Standard errors in parentheses.

Table S40: **Models examining whether student speech is classified as hate speech.** Student Experiment by Principle among Those More Sympathetic to Israelis. DV: Hate Speech

	Particularistic		Universalistic	
	(1)	(2)	(1)	II
Intercept	0.25***	0.31**	0.19***	-0.01
	(0.06)	(0.11)	(0.05)	(0.12)
Target Group: Muslim	-0.16**	-0.16**	-0.15**	-0.14**
	(0.05)	(0.05)	(0.05)	(0.05)
Statement: Oppressive Ideology	0.29***	0.29***	0.12+	0.12+
	(0.09)	(0.08)	(0.06)	(0.07)
Statement: Shady Practices	0.27**	0.28**	0.16*	0.21**
	(0.09)	(0.09)	(0.08)	(0.08)
Statement: World Better Off Without Them	0.65***	0.66***	0.55***	0.55***
	(0.07)	(0.07)	(0.07)	(0.07)
Statement: Root of All Evil	0.70***	0.70***	0.68***	0.70***
	(0.07)	(0.07)	(0.07)	(0.07)
Num.Obs.	259	259	286	286
R2	0.302	0.362	0.314	0.351
Controls	No	Yes	No	Yes

+ p <0.1, * p <0.05, ** p <0.01, *** p <0.001
Standard errors in parentheses.

Table S41: **Models examining support for firing professors based on real statements.** Statement Experiment. DV=Fire Professor

	I	II
Intercept	0.18***	0.17***
	(0.01)	(0.03)
Statement: DEI	-0.01	-0.01
	(0.02)	(0.02)
Statement: Kaepernick	0.36***	0.36***
	(0.02)	(0.02)
Statement: White Genocide	0.36***	0.36***
	(0.02)	(0.02)
Num.Obs.	2966	2966
R2	0.144	0.159
Controls	No	Yes

+ p <0.1, * p <0.05, ** p <0.01, *** p <0.001

Table S42: **Models examining responses to real professor statements.** Publish Statement Experiment. DV=Publish Statement

	I	II
Hamas's attack on Israel on October 7	-0.09**	-0.10***
	(0.03)	(0.03)
Israel's invasion of Rafah	-0.09**	-0.09***
	(0.03)	(0.03)
the 2016 gay nightclub shooting	0.02	0.01
	(0.03)	(0.03)
the Russia-Ukraine conflict	-0.07*	-0.08**
	(0.03)	(0.03)
Num.Obs.	2966	2966
R2	0.009	0.100
Controls	No	Yes

+ p <0.1, * p <0.05, ** p <0.01, *** p <0.001

Table S43: **Models examining support for allowing speakers to hold events on campus.** Events Experiment. DV=Allow Event

	I	II
Enrique Tarrio	-0.05	-0.05
	(0.04)	(0.04)
George Soros	0.38***	0.38***
	(0.03)	(0.03)
Ismail Haniyeh	-0.11**	-0.11**
	(0.04)	(0.04)
Itamar Ben-Gvir	-0.01	-0.01
	(0.04)	(0.04)
Mahmoud Abbas	0.22***	0.22***
	(0.03)	(0.03)
Rupert Murdoch	0.21***	0.22***
	(0.03)	(0.03)
Samuel Miller	0.15***	0.14***
	(0.04)	(0.03)
Num.Obs.	2966	2966
R2	0.107	0.128
Controls	No	Yes

+ p <0.1, * p <0.05, ** p <0.01, *** p <0.001

Table S44: **Models examining support for allowing speakers to hold events on campus.** Events Experiment. DV=Allow Event

	I	II
Target: Israeli	-0.06*	-0.06*
	(0.03)	(0.02)
Target: Left	0.21***	0.21***
	(0.02)	(0.02)
Target: Right	0.02	0.03
	(0.02)	(0.02)
More Objectionable	-0.21***	-0.21***
	(0.02)	(0.02)
Num.Obs.	2966	2966
R2	0.092	0.113
Controls	No	Yes

+ p <0.1, * p <0.05, ** p <0.01, *** p <0.001

Additional Supplementary Files

None.
